# Widespread *Wolbachia* infection in an insular radiation of damselflies (Odonata, Coenagrionidae)

**DOI:** 10.1038/s41598-019-47954-3

**Published:** 2019-08-15

**Authors:** M. O. Lorenzo-Carballa, Y. Torres-Cambas, K. Heaton, G. D. D. Hurst, S. Charlat, T. N. Sherratt, H. Van Gossum, A. Cordero-Rivera, C. D. Beatty

**Affiliations:** 1 ECOEVO Lab, EE Forestal, Campus Universitario A Xunqueira s/n, 36005 Pontevedra, Spain; 2 Institute of Integrative Biology, University of Liverpool, Crown Street, L69 7ZB Liverpool, United Kingdom; 3 Departamento de Biología y Geografía, Facultad de Ciencias Naturales y Exactas, Universidad de Oriente, Avda. Patricio Lumumba s/n, Santiago de Cuba, 90500 Cuba; 4 Université de Lyon, Université Lyon 1, CNRS, UMR 5558, Laboratoire de Biométrie et Biologie Evolutive, 16, rue Raphael Dubois, 69622 Villeurbanne, France; 5 Department of Biology, Carleton University 1125 Colonel By Drive, Ottawa, ON K1S 5B6 Canada; 6 Evolutionary Ecology Group, University of Antwerp, Campus Drie Eiken, Universiteitsplein 1 D.D.136 2610, Wilrijk Antwerp, Belgium; 7 Department of Ecology & Evolutionary Biology, Cornell University, E149 Corson Hall, 215 Tower Road, Ithaca, NY 08053 United States of America

**Keywords:** Evolutionary ecology, Speciation

## Abstract

*Wolbachia* is one of the most common endosymbionts found infecting arthropods. Theory predicts symbionts like *Wolbachia* will be more common in species radiations, as host shift events occur with greatest frequency between closely related species. Further, the presence of *Wolbachia* itself may engender reproductive isolation, and promote speciation of their hosts. Here we screened 178 individuals belonging to 30 species of the damselfly genera *Nesobasis* and *Melanesobasis* — species radiations endemic to the Fiji archipelago in the South Pacific — for *Wolbachia*, using multilocus sequence typing to characterize bacterial strains. Incidence of *Wolbachia* was 71% in *Nesobasis* and 40% in *Melanesobasis*, and prevalence was also high, with an average of 88% in the *Nesobasis* species screened. We identified a total of 25 *Wolbachia* strains, belonging to supergroups A, B and F, with some epidemic strains present in multiple species. The occurrence of *Wolbachia* in both males and females, and the similar global prevalence found in both sexes rules out any strong effect of *Wolbachia* on the primary sex-ratio, but are compatible with the phenotype of cytoplasmic incompatibility. *Nesobasis* has higher species richness than most endemic island damselfly genera, and we discuss the potential for endosymbiont-mediated speciation within this group.

## Introduction

*Wolbachia* is a genus of endosymbiotic α-Proteobacteria, which infects a wide range of hosts, including arthropods and filarial nematodes^1^. Among the arthropoda, studies have estimated *Wolbachia* infects between 40%^2^ and 52%^3^ of species (including arachnids and crustaceans, but predominantly insects). A recent study on aquatic insects has estimated that 52% of these carry *Wolbachia*, a figure comparable to that found in terrestrial insects^4^. In most cases however, a minority (<10%) of individuals within species are infected^5^. *Wolbachia* are categorised as reproductive parasites, *i.e.* maternally inherited microorganisms that manipulate the reproduction of their hosts in ways that enhance the production or the survivorship of infected females, hence increasing their own fitness. Reproductive alterations induced by *Wolbachia* include the feminization of chromosomally male embryos, killing of male hosts during embryogenesis, induction of thelytokous parthenogenesis, and cytoplasmic incompatibility (CI). The latter prevents successful mating between infected males and uninfected females, or between individuals infected with different, incompatible *Wolbachia* strains^1^. Beyond these reproductive manipulations, *Wolbachia* can also evolve mutualistic associations with their hosts, including facultative relationships that increase host fecundity or host survival/longevity, provide nutritional provisioning, or protect hosts against pathogenic attacks. In some cases hosts have evolved complete dependence upon *Wolbachia*^6,7^.

Vertical transmission of *Wolbachia* from mother to offspring occurs through the germline and somatic stem cell niches^8^. On a longer evolutionary scale, vertical transmission can be identified by a shared phylogeny between the endosymbionts and their hosts. However, this pattern is usually not observed for *Wolbachia*, reflecting a process in which vertical transmission within species combines with occasional horizontal transmission between species^9,10^. In order to gain evidence for either strict vertical inheritance or occasional horizontal transfer in a particular host clade, one can compare the phylogenetic tree of the symbionts to that of the hosts. This approach also provides hints into the possible nature of the interaction, since obligate mutualists tend to be strictly vertically transmitted, whereas facultative symbionts are more often transferred horizontally^11^. Approaches like these have been used, for example, to examine the sources of endosymbiont infections in the wasp genus *Nasonia*, which comprises four species carrying multiple *Wolbachia* strains^12^. They have also allowed for the production of global estimates of the rates of *Wolbachia* loss and acquisition^10^.

In the present study, we examine the patterns of *Wolbachia* incidence in a radiation of island damselflies. It has been suggested that species radiations provide the conditions under which symbiont incidence may rise to very high levels^13^. This theory derives from the increased likelihood of symbionts undergoing a host shift event to closely related host species, which is the outcome of a combination of high contact rates (*e*.*g*. through hybridization and/or shared parasites) introducing infections across the radiation, with the close physiological and ecological similarity of the hosts making the symbiont more likely to be compatible with the new host species. Further to this, the presence of diverse symbionts may potentiate speciation in species radiations, through the spread of symbiont strains that encode CI in their hosts, and which may produce barriers to gene flow^14–16^.

**Figure 1:**
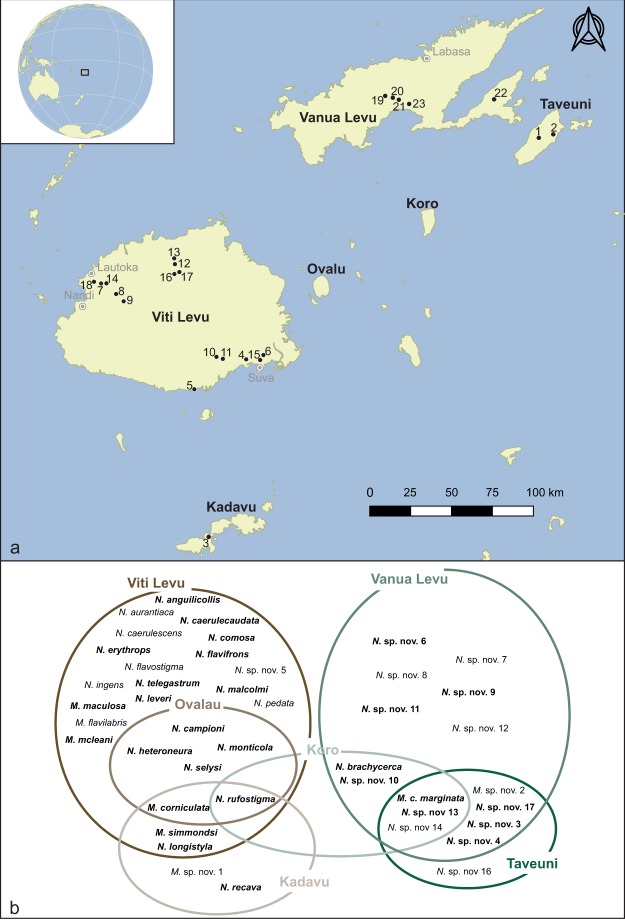
(**a**) Map of the Fiji islands, showing the locations where the *Nesobasis* and *Melanesobasis* species included in the present study were collected. The numbers shown on the map correspond with the sampling locations as follows: 1 – Mt. Devo; 2 – Bouma Track Upper Falls; 3 – SW Namalata Village; 4 – Waivudawa Creek; 5 – Ocean Pacific Resort Trail; 6 – Colo-i-Suva; 7 – Abaca Road 3; 8 – Sabeto River; 9 – Vaturu Dam Road 2; 10 – Namosi Road 6; 11 – Wainikovu; 12 – Korowaiwai; 13 – Waikubukubu; 14 – Vereni Falls; 15 – Vaqo Creek; 16 – Nukunuku; 17 – Qualiwana Tributary; 18 – Abaca Road 2; 19 – Raviravi Creek; 20 – Saivou Village; 21 – Lomaloma Falls; 22 – Niuwauvudi Creek; 23 – Sauvuqoro Creek. For details on which samples were collected at each site, see Suppl. Information Table S1. **(b)** Venn diagram illustrating the distribution of all the known *Melanesobasis* and *Nesobasis* species among the Fiji islands. The species included in the present study appear in bold.

*Nesobasis* is one of the most species-rich genera of Odonata (dragonflies and damselflies) found in oceanic islands. It is endemic to the Fiji archipelago, and currently, there are 21 *Nesobasis* species described, and 15 more awaiting description^17^ (N. Donnelly pers. comm. and authors’ own data, see Fig. 1a). Only the genus *Megalagrion* in the Hawaiian Islands has a comparable level of species diversity for an insular group of odonates^18–20^. The genus *Melanesobasis* includes a total of seven described species and one sub-species, with another two species currently undescribed^21^; seven of these are found exclusively in Fiji, while *M*. *bicellulare* occurs on Maewo in Vanuatu. Some species of *Nesobasis* display female-biased sex ratios at oviposition sites along forested streams^17,22^. Males of several species have been found to be territorial, defending positions along streams and approaching passing females; and females, even in those species where males are rare, do not defend territories^22^.

Species within *Nesobasis* show striking morphological diversity, with large differences in coloration and size among species^23^ as well as elaborate secondary reproductive structures in males and females^17^. A recent molecular phylogeny and biogeographical analysis of *Nesobasis* and *Melanesobasis*^23^ found that, while current species distributions fall into two main assemblages associated with the two largest islands in the archipelago (Viti Levu and Vanua Levu; see Fig. 1b) some dispersal has happened over evolutionary time, such that these assemblages do not form distinct phylogenetic clades. While dispersal seems to have been involved in speciation in *Nesobasis*, a large number of speciation events appear to have taken place within a single island, an interesting pattern considering this group of species has very little ecological diversification, with many species occurring sympatrically in forested stream habitats. Diversification rates also appear to have increased significantly over time within these taxa, a pattern associated in some models with non-ecological speciation^24,25^.

In a preliminary screening, some species of *Nesobasis* were found to be infected by *Wolbachia*^22^. Such a large radiation showing *Wolbachia* infections provides a relevant system to gain insights into the mode of symbiont transfer within clades, and to investigate any potential contribution of *Wolbachia* to speciation. The presence of multiple, closely related species of *Nesobasis* may enable endosymbiont transfer between species through hybridization, whereas the presence of ectoparasitic mites, commonly found on odonates, could represent vectors of genuine horizontal transfer events. Here, we examine the pattern of symbiont incidence in these damselfly genera. We report on the results of a *Wolbachia* screening for a total of 178 individuals from 25 *Nesobasis* and 5 *Melanesobasis* species. We used a multilocus sequence typing (MLST) approach to characterize the strains found infecting these damselflies, sequenced the hosts nuclear and mitochondrial DNA to provide further insights into the evolutionary history of these host-symbiont associations, and examined correlations between the host and endosymbiont phylogenies.

## Results

### Incidence and prevalence of *Wolbachia* infections in Nesobasis and Melanesobasis

**Figure 2:**
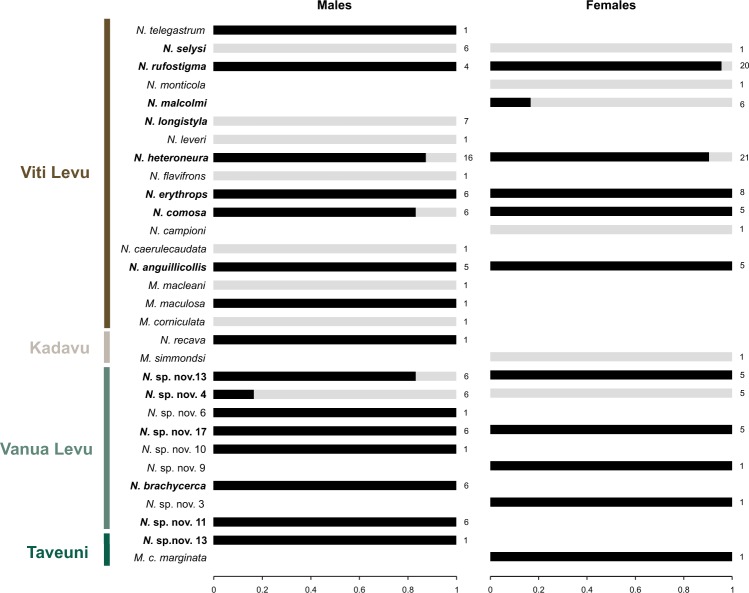
Infection rates of *Wolbachia* in the 30 *Nesobasis* and *Melanesobasis* species from Fiji archipelago that were screened for the present study. Black bars show the infection rates per species and host sex. Numbers at the right of each bar indicate the number of individual of each species and sex included in the screening. Note that for some species, only individuals of one sex were available for screening. Highlighted in bold are the species with >5 individuals screened for infection that were used for prevalence calculations (see Table 1).

A total of 178 individuals belonging to 25 *Nesobasis* and 5 *Melanesobasis* species, representing respectively 69.45% and 71.43% of the described species diversity in each genus, were screened for the presence of *Wolbachia*. The incidence of infection was 72% in *Nesobasis* (18 infected species out of the 25 species screened) and 40% in *Melanesobasis* (2 out of 5 species screened were found to be infected). Considering only those species for which at least 5 individuals could be screened (all of them within *Nesobasis*), the prevalence of *Wolbachia* infection ranged from 9.1% to 100% across species (Table 1, Fig. 2). *Wolbachia* was observed in species with both normal and female-biased sex ratios in the field (Table 1), and no correlation between *Wolbachia* infection status and host sex was observed (Fig. 2). Pooling specimens from all species, the total prevalence of *Wolbachia* was 80.5% in males and 70.3% in females and this difference was not significant (χ^2^ = 2.45; df = 1; P = 0.117). Interestingly, a significant difference in incidence of *Wolbachia* infection was found between the two island groups, with a higher proportion of Vanua Levu than Viti Levu species of *Nesobasis* being infected with *Wolbachia* (100% of species from Vanua Levu *vs*. 47.37% of species from Viti Levu; χ^2^ = 5.93; df = 1; P = 0.015).

**Table 1:** Summary of prevalence of *Wolbachia* infection and *Wolbachia* supergroups identified in the *Melanesobasis* and *Nesobasis* species screened for this study.

Island Group	Species	No of individuals screened			% of infected individuals			No of MLST typed individuals	*Wolbachia* supergroups
		Males	Females	Total	Males	Females	Total		
Viti Levu	Nesobasis telegastrum*	1	0	1	—	—	—	1	F
	*N*. *selysi*	6	1	7	0%	0%	0%	n.a.	n.a.
	*N*. *rufostigma*	4	19	23	100%	94.7%	95.7%	2	A, F
	*N*. monticola*	0	1	1	—	—	—	n.a.	n.a.
	*N*. *malcolmi*	0	6	6	n.a.	17%	17%	1	A, B
	*N*. *longistyla*	7	0	7	0%	n.a.	0%	n.a.	n.a.
	*N*. leveri*	1	0	1	—	—	—	n.a.	n.a.
	*N*. *heteroneura*	16	21	37	93.8%	90.5%	91.9%	2	A, B
	*N*. flavifrons*	1	0	1	-	-	-	n.a.	n.a.
	*N*. *erythrops*	6	8	14	100%	100%	100%	2	A, B
	*N*. *comosa*	7	5	12	71%	100%	83.3%	2	A, B
	*N*. campioni*	0	1	1	—	—	—	n.a.	n.a.
	*N*. caerulecaudata*	1	0	1	—	—	—	n.a.	n.a.
	*N*. *anguillicolis*	5	5	10	100%	100%	100%	2	A, F
	Melanesobasis mcleani*	1	0	1	—	—	—	n.a.	n.a.
	*M*. maculosa*	1	0	1	—	—	—	1	A
	*M*. corniculata*	1	0	1	—	—	—	n.a.	n.a.
	*N*. recava*	1	0	1	—	—	—	1	F
	*M*. simmondsi*	0	1	1	—	—	—	n.a.	n.a.
Vanua Levu	*N*. sp. nov. 13	7	5	12	85.7%	100%	91.7%	3	A, F
	*N*. sp. nov. 4	6	5	11	17%	0%	9.1%	1	B
	*N*. sp. nov. 6*	1	0	1	—	—	—	1	A,F
	*N*. sp. nov. 17	6	5	11	100%	100%	100%	2	B
	*N*. sp. nov. 10*	1	0	1	—	—	—	1	F
	*N*. sp. nov. 9*	0	1	1	—	—	—	1	A,F
	*N*. *brachycerca*	6	0	6	100%	n.a.	100%	2	A, B, F
	*N*. sp. nov. 3*	0	1	1	—	—	—	1	F
	*N*. sp. nov. 11	6	0	6	100%	n.a.	100%	2	A, B
	*M*. *c*. marginata*	0	1	1	—	—	—	1	A

Species of *Nesobasis* reported in the literature as having female-biased sex ratios in the field appear highlighted in bold. Species with less than five specimens included in the screening, and hence not used for prevalence calculations, are marked with an asterisk. Additional information on the strains found in each of the MLST typed individuals is given in the Suppl. Information Table S1.

### *Wolbachia* MLST characterization and strain distribution

**Figure 3:**
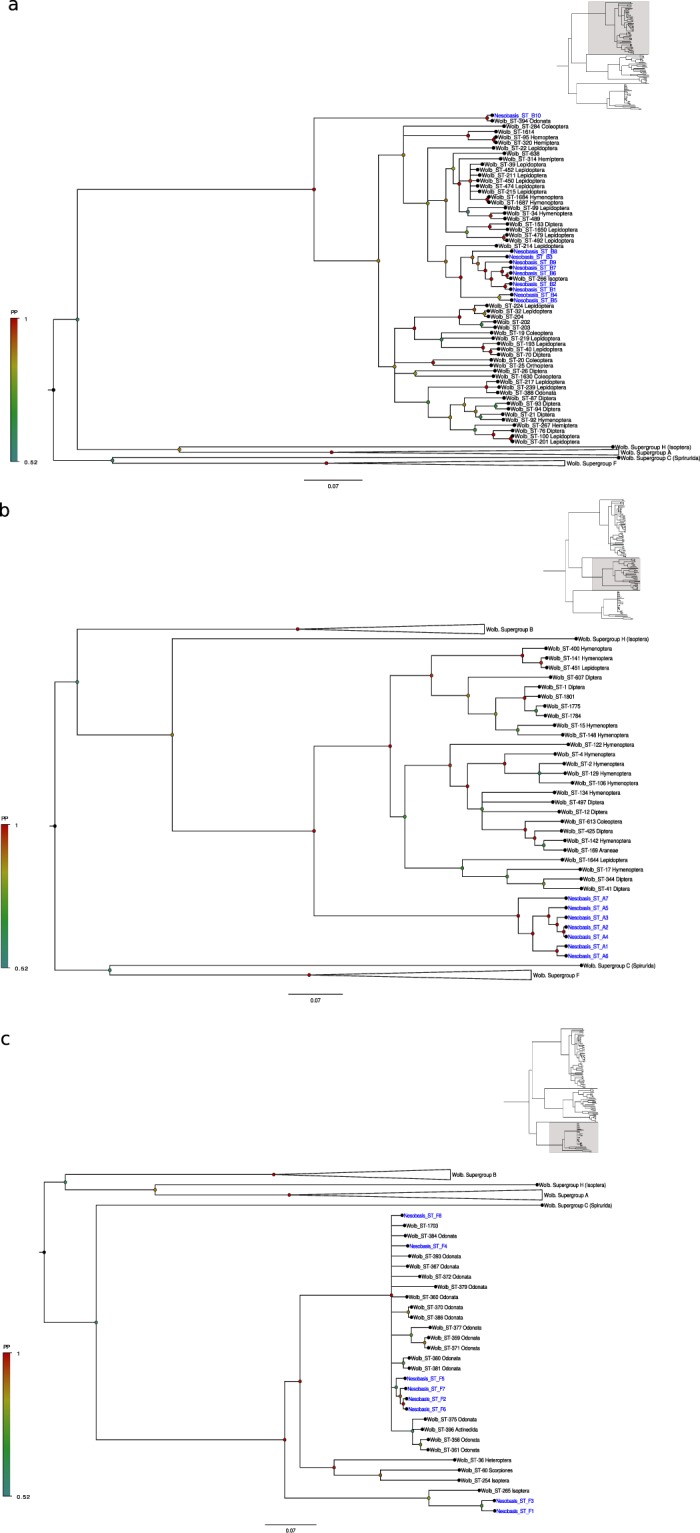
Midpoint rooted phylogenetic tree showing the results of the analysis of a 2,084 bp alignment of five concatenated *Wolbachia* MLST gene sequences (*coxA*, *gatB*, *hcpA*, *ftsZ* and *fbpA*), using ClonalFrame. Each subtree depicts the relationships between *Wolbachia* strains belonging to supergroup B (**a**), supergroup A (**b**) and supergroup F (**c**). *Wolbachia* strains found infecting the *Nesobasis* and *Melanesobasis* species screened in our study appear in blue, whereas strains in black correspond to *Wolbachia* allelic profiles downloaded from the MLST database (http://pubmlst.org/*wolbachia*/, see Suppl. Information Table 4). Nodes are colored according to their posterior probability values, as depicted in the tree legend.

Twenty-nine individuals belonging to the 20 infected *Nesobasis* and *Melanesobasis* species were selected for MLST genotyping of the *Wolbachia*. A total of 34 alleles from the genes *gatB*, *coxA*, *hcpA* and *fbpA* were identified, of which 12 were new to the MLST database (Table 2; http://pubmlst.org/*wolbachia*). This allelic diversity represented 25 unique *Wolbachia* strains (Table 2), which were identified by the ClonalFrame analyses as belonging to supergroups B, A and F (Fig. 3).

**Table 2:** Allelic profiles of the *Wolbachia* infections found in the *Melanesobasis* and *Nesobasis* species screened for this study.

Strain Name	Supergroup	gatB	coxA	hcpA	ftsZ	fbpA	Host species
Nesobasis_ST-A1	A	248*	?	275*	unique (near to 218)	416*	*Melanesobasis corniculata marginata*, *M*. *maculosa*, *Nesobasis heteroneura*, *N*. *comosa*, *N*. *malcolmii*, *N*. sp. nov. 11, *N*. *erythrops*, *N*. *brachycerca*, *N*. *rufostigma*
Nesobasis_ST-A2	A	82	?	275*	unique (near to 218)	125	*N*. sp. nov. 13, *N*. *rufostigma*, *N*. sp. nov. 6
Nesobasis_ST-A3	A	unique (near to 82)	?	275*	unique (near to 218)	unique (near to 125)	*N*. *rufostigma*, *N*. *brachycerca*
Nesobasis_ST-A4	A	249*	?	275*	unique (near to 218)	125	*N*. *anguillicolis*
Nesobasis_ST-A5	A	unique (near to 249)	?	275*	unique (near to 218)	n.a.	*N*. sp. nov. 11
Nesobasis_ST-A6	A	unique (near to 248)	?	275*	unique (near to 218)	n.a.	*N*. sp. nov. 6
Nesobasis_ST-A7	A	250*	231*	275*	?	416*	*N*. sp. nov. 9
Nesobasis_ST-B1	B	9	147	263	unique (near to 125)	9	*N*. *brachycerca*
Nesobasis_ST-B2	B	9	?	unique (near to 263)	unique (near to 125)	9	*N*. *brachycerca*
Nesobasis_ST-B3	B	9	147	277*	unique (near to 125)	9	*N*. sp. nov. 11
Nesobasis_ST-B4	B	9	?	277*	unique (near to 125)	9	*N*. *brachycerca*
Nesobasis_ST-B5	B	9	230*	277*	unique (near to 125)	9	*N*. sp. nov. 11, *N*. sp. nov. 17
Nesobasis_ST-B6	B	?	231*	263	?	416*	*N*. *heteroneura*, *N*. *comosa*
Nesobasis_ST-B7	B	?	unique (near to 147)	263	?	416*	*N*. *heteroneura*
Nesobasis_ST-B8	B	9	36	276*	unique (near to 125)	9	*N*. *erythrops*
Nesobasis_ST-B9	B	9	231*	276*	unique (near to 125)	416*	*N*. *malcolmii*
Nesobasis_ST-B10	B	9	107	29	?	417*	*N*. sp. nov. 4
Nesobasis_ST-F1	F	82	232*	263	?	125	*N*. *brachycerca*
Nesobasis_ST-F2	F	249*	147	278*	unique (near to 132)	125	*N*. *recava*
Strain Name	Supergroup	gatB	coxA	hcpA	ftsZ	fbpA	Host species
Nesobasis_ST-F3	F	unique (near to 130)	232*	263	?	125	*N*. *brachycerca*
Nesobasis_ST-F4	F	?	147	263	?	?	*N*. sp. nov. 13, *N*. sp. nov. 6, *N*. *rufostigma*
Nesobasis_ST-F5	F	?	147	263	?	6	*N*. *anguillicolis*
Nesobasis_ST-F6	F	82	147	263	unique (near to 132)	125	*N*. sp. nov. 13
Nesobasis_ST-F7	F	82	147	263	?	125	*N*. sp. nov. 3
Nesobasis_ST-F8	F	?	147	263	?	125	*N*. *telegastrum*, *N*. sp. nov. 10

Listed are: the name given to each strain, the supergroup to which it belongs (according to the results of the ClonalFrame analyses), the allele number for each of the MLST genes, and the species in which that strain has been found. “Unique” alelles refer to those that have not been reported to the MLST database; and “near to” refers to the closest match for these alleles in the MLST database. “?” Indicates that no sequence could be obtained for a particular MLST locus. “*” Indicates the alleles that were new to the MLST database.

*Wolbachia* strains belonging to supergroup B (n = 10) were found in 8 of the *Nesobasis* species analyzed (Tables 1 and 2). Strains B1 to B9 grouped together with high support in the ClonalFrame analysis (Fig. 3a), with B strains found infecting Isoptera and Lepidoptera as the closest relatives. Strain B10 grouped together with strain 394 from the MLST database, which infects the dragonfly species *Crocothemis servilia*. With the exception of strains B5 and B6, which were found in 2 *Nesobasis* species each; the remaining B strains were each found in a single *Nesobasis* species (Table 2).

Strains belonging to supergroup A (n = 7) clustered together with high support in the ClonalFrame analysis, forming a monophyletic clade, well differentiated from the other A strains from the MLST database included in the analysis (Fig. 3b). Supergroup A strains were the most common in our sample, being found in 11 *Nesobasis* species, and the 2 *Melanesobasis* species (*i*.*e*. in 13 out of the 20 infected species*;* Table 1). From these, strain A1 seems to be epidemic, as it has been found in 7 of the *Nesobasis* and in the two *Melanesobasis* infected species. Strain A2 was found in 3 *Nesobasis* species; strain A3 was found in two *Nesobasis* species, and the others (strains A4 to A7) were found in a single *Nesobasis* species each (Table 2). Notably, the A supergroup had not been identified in earlier surveys of odonates, suggesting *Wolbachia* diversity is not randomly distributed in this insect order.

Strains falling in the F supergroup (n = 8) were found in 10 *Nesobasis* species (Tables 1 and 2). Most of the F strains appeared as closely related to other F strains found infecting odonates^26^ (Fig. 3c), whereas strains F1 and F3 group together with *Wolbachia* strain 265 (occurring in Isoptera) as their closest relative. With the exception of strains F4 and F8, which were found in 3 and 2 *Nesobasis* species respectively, F strains were each detected in a single *Nesobasis* species.

Occurrence of coinfecting *Wolbachia* lineages was common in our samples: 11 out of the 20 infected species showed infection by more than one *Wolbachia* strain (Table 1). Co-occurrence of A and B *Wolbachia* strains was observed in individuals belonging to 6 species: *Nesobasis sp*. *nov*. *11* (n = 2), *N*. *brachycerca* (n = 1), *N*. *comosa* (n = 1), *N*. *erythrops* (n = 2), *N*. *heteroneura* (n = 1), and *N*. *malcolmi* (n = 1). Co-infection by A and F strains was found in individuals from 4 species: *N*. *anguillicolis* (n = 2), *N*. *rufostigma* (n = 2), *N*. *sp*. *nov*. *6* (n = 1) and *N*. *sp*. *nov*. *13* (n = 1). Finally, co-occurrence of B and F supergroups was found in one specimen of *N*. *brachycerca*.

Single *Wolbachia* infections were found in individuals belonging to 10 species. Individuals belonging to *Melanesobasis corniculata marginata* (n = 1), *M*. *maculosa* (n = 1), *N*. *comosa* (n = 1), *N*. *heteroneura* (n = 1) and *N*. *sp*. *nov*. *9* (n = 1) showed infection by a single *Wolbachia* A strain. *N*. *sp*. *nov*. *17* (n = 2) and *N sp*. *nov*. *4* (n = 1) showed single B strain infection; and finally, *N*. *recava* (n = 1), *N*. *telegastrum* (n = 1) *N*. *sp*. *nov*. *3* (n = 1), *N*. *sp*. *nov*. *10* (n = 1) and *N*. *sp*. *nov*. *13* (n = 2), were infected by F *Wolbachia* strains (see Table 1 and Suppl. Information Table S1 for details on strains infecting each species/individual).

### Phylogenetic relationships among host species

**Figure 4:**
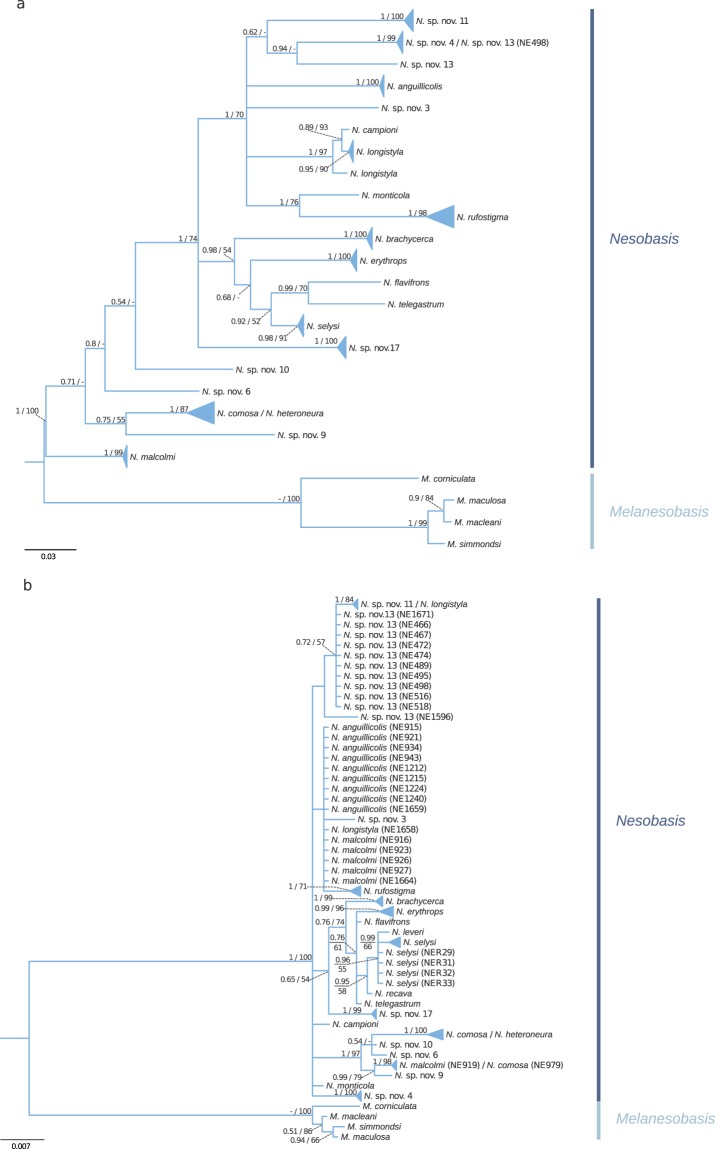
Midpoint rooted Bayesian phylograms, showing the results of the analysis of a mitochondrial (*COI*; **a**) and a nuclear (*PRMT*; **b**) locus for the *Nesobasis* and *Melanesobasis* species included in this study. Values above branches indicate phylogenetic support values (BI/ML). Only values above 50% bootstrap support and 0.5 pp are shown.

For each of the markers used to infer the host evolutionary history, phylogenetic trees obtained with BI and ML methods were congruent. The genera *Nesobasis* and *Melanesobasis* were both recovered as monophyletic groups with high support using both *COI* and *PRMT* loci (see Fig. 4). In the case of *Nesobasis*, the monophyly of some species was also well supported regardless of the marker used (*e*.*g*. *N*. *rufostigma*, *N*. *selysi*, *N*. *brachycerca*, *N*. *erythrops*, *N*. *comosa*/*N*. *heteroneura* and *N*. sp. nov. 17). For other species included in the study, monophyly could not be tested as they were represented by a single individual (*e*.*g*. *N*. sp. nov. 6, *N*. sp. nov. 10 or *N*. sp. nov. 3). Some instances of unresolved or poorly supported nodes were observed, especially for the *PRMT* locus, which could be due to incomplete lineage sorting, or a rapid and recent species radiation^23^. Discrepancies between nuclear and mitochondrial phylogenies may also stem from introgression of mitochondria driven by symbionts^27^. Below we explore the congruence (or lack thereof) between our *COI* and *PRMT* phylogenies.

The *“erythrops A”* clade from Beatty *et al*.^23^, which includes the species *N*. *brachycerca*, *N*. *erythrops*, *N*. *flavifrons*, *N*. *telegastrum*, *N*. *recava* and *N*. *selysi*, is well supported in all analyses and by all markers (see Fig. 4). However, the other major *Nesobasis* clades as described by Beatty *et al*.^23^ were in some cases not well supported or showed incongruences between nuclear and mitochondrial markers. Such is the case of the *“comosa”* group *sensu* Beatty *et al*.^23^. This clade includes the species *N*. *heteroneura*, *N*. *comosa*, *N*. sp. nov. 6, *N*. sp. nov. 10 and *N*. *malcolmi*; it appears in our case reasonably well supported by the *PRMT* locus (Fig. 4b). However, this clade appears to be paraphyletic in the *COI* tree (Fig. 4a). Interestingly, while the individuals of *N*. *malcolmi* form a monophyletic and well supported group in the *COI* tree (see Fig. 4a), this species is not recovered as monophyletic in the *PRMT* phylogeny, with one individual (NE919) found within the “*comosa*” clade, and the remaining *N*. *malcolmi* grouping together with *N*. sp. nov. 3, *N*. *anguillicolis* and a *N*. *longistyla* individual (all species belonging to the “*erythrops B/longistyla*” clade *sensu* Beatty *et al*.^23^). This group appears as well supported by the *COI* locus (BI posterior probability = 1 and ML bootstrap value = 70%; Fig. 4a), and includes also the species *N*. *monticola*, which was not included in Beatty *et al*.^23^. However, in the *PRMT* phylogeny, most species of this clade split in two main groups: on one side, *N*. sp. nov. 13, *N*. sp. nov. 11 and the majority of the *N*. *longistyla* individuals make a monophyletic group with a relatively good support (BI posterior probability = 0.72 and ML bootstrap value = 57%; Fig. 4b); while *N*. *rufostigma*, *N*. *anguillicolis*, *N*. sp. nov. 3 and one *N*. *longistyla* specimen group separately with the majority of the *N*. *malcolmi* individuals, as mentioned above, although this cluster is not supported by any of the analyses (Fig. 4b).

Despite our results being largely congruent with the recently established phylogeny of *Nesobasis*^23^, the observed differences stress the need to include a higher number of molecular markers, along with increasing the numbers of specimens in order to obtain a more complete picture of these species’ relationships, and thus distinguish phylogenetic artefacts from true heterogeneity in the evolutionary histories of different genomic regions. In particular, most of the observed differences stem from the use of *PRMT* in our study. Being an intron, this marker has a higher rate of evolution than protein-coding markers, therefore being more suitable for resolving low taxonomic relationships. Including *PRMT* together with additional nuclear markers in our study would probably help to better account for phylogenetic discordance across the genome due to lineage sorting or interspecific hybridization.

### Cophylogenetic analyses

**Figure 5:**
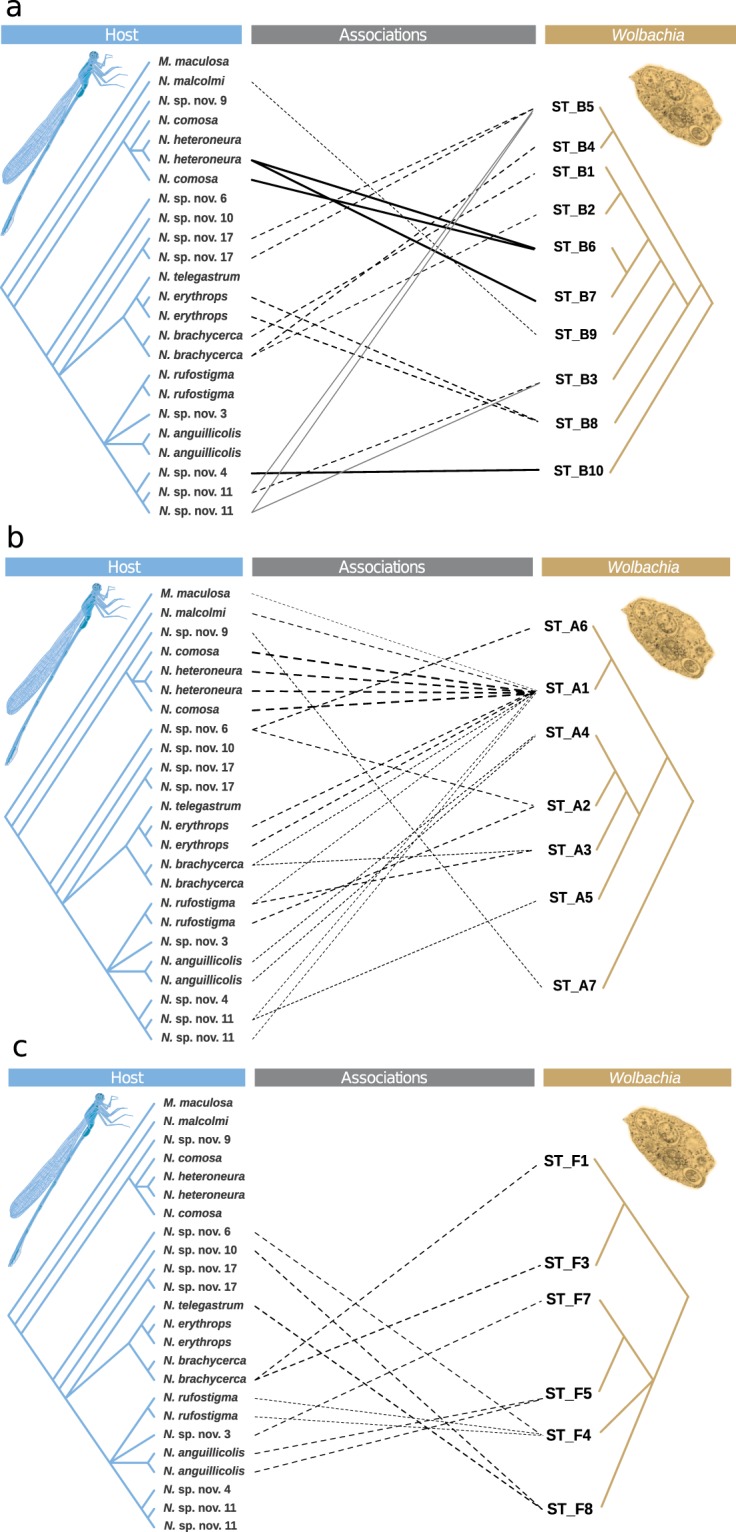
Tanglegrams depicting the associations between *Wolbachia* strains belonging to supergroup B (**a**), supergroup A (**b**), supergroup F (**c**) and their *Nesobasis* and *Melanesobasis* damselfly hosts. Solid link lines represent associations that contributed significantly to the overall congruence between both topologies. Gray link lines constitute significant associations in both ParaFitLink1 and ParafitLink2 tests (α = 0.01). Dashed lines represent associations whose 95% confidence intervals for the squared residual values in the Procrustean Approach to Cophylogeny analysis (PACo) were below the median value. The thickness of the lines is inversely proportionate to the squared residual values of each association and therefore is suggestive of the contribution of each association to the overall congruence detected in the PACo analysis (thicker lines indicate a higher contribution).

The results of ParaFit and PACo analyses suggest there is some level of topological congruence between the phylogenies of *Wolbachia* and their damselfly hosts (ParaFit-Global = 0.036, p = 0.002; PACo m^2^_XY_ = 0.855, p < 0.001). In addition, PACo results suggest that host and *Wolbachia* are not randomly associated, although it is unclear which of the individual associations contributed to the overall congruence, because the results of ParaFitLink1 and ParaFitLink2 tests differed from those of the squared residual values of PACo (see Fig. 5, Suppl. Information Fig. S1, Suppl. Information Table S5).

The analysis with Jane found a total of 1,000 solutions that explain the association between *Wolbachia* and their hosts, all of them with the same total cost (93). These were reduced to 12 after compressing isomorphic solutions. These solutions can be considered sufficiently robust because their total cost was smaller than the cost of all simulated samples. The most frequent events found were: loss (40), followed by failure to diverge (24), duplication and host switch (*i*.*e*. horizontal transfer) (10), duplication (7) and co-speciation (5) (see Suppl. Information Fig. S2). The preponderance of events where the speciation of the hosts was not followed by the divergence of *Wolbachia* (duplication, duplication and host switch, failure to diverge) suggests that these host-symbiont associations are not stable through evolutionary time. The high incidence of *Wolbachia*, despite the high frequency of loss events, suggests that reinfections have been frequent during the evolutionary history of associations between these bacteria and their damselfly hosts. Some of these reinfections could take place via horizontal transmission (*i*.*e*. duplication and host switch) between hosts of the same or a different genus, or through hybridization (see Suppl. Information Fig. S2). Regarding the co-speciation events, only the one involving the *Nesobasis* species *N*. sp. nov. 11 (individuals NE0613, NE0638) and *N*. sp. nov. 4 (NE0455) and the *Wolbachia* strains ST_B10, ST_B3 and ST_B5, included associations of high contribution to the overall congruence between topologies detected by ParaFit and PACo (see Fig. 5, Suppl. Information Figs S1 and S2, and Suppl. Information Table S5).

## Discussion

Theory predicts that endosymbionts may become more common in groups of closely related species, as the probability of symbiont transmission between host species declines with increasing genetic distance between hosts. In other words, a high incidence of infection is expected in clades undergoing radiation^13^. Consistent with this, our results indicate that the incidence of *Wolbachia* in *Nesobasis* species is among the highest reported in the literature for this endosymbiont. While previous screenings using methodologies similar to ours (*i*.*e*. standard PCR screening) have shown an average *Wolbachia* incidence of 20% in arthropods (mainly insects)^28,29^, we have found that 72% of the *Nesobasis* species screened are infected by this endosymbiont. This value is much higher than previously estimated incidences for other terrestrial and aquatic insect species^4,5^. The proportion of infected individuals within species was also higher than reported for other insect orders^5^, with only one species (*N*. sp. nov. 4) having fewer than 10% of screened individuals infected (see Table 1). Only two out of five *Melanesobasis* species were found infected, suggesting a possibly lower incidence in this genus than in *Nesobasis*, although a larger sample size would be needed to confirm this trend. It is important to note that our *Wolbachia* incidence estimation is conservative, since only one individual was available for many of the screened species (see Fig. 2). Together with the possibility of a false negative PCR, this limits our ability to detect low prevalence infections.

We used an MLST approach to analyse if the high incidence of *Wolbachia* was associated with highly diverse *Wolbachia* infections, or whether the high incidence of *Wolbachia* was associated with fewer strains showing epidemic spread across the clade, as predicted by Engelstädter and Hurst^13^ for adaptive radiations. We found a remarkably high level of diversity of *Wolbachia* in our sample: 25 unique strains were identified in the 29 *Melanesobasis* and *Nesobasis* individuals investigated. Nevertheless, particular epidemic strains were found in multiple species (*e*.*g*. strain A1, Fig. 5b), thus implying that host shifts within the clade explain part of the high incidence. Acquisition from more distant hosts also appears to have contributed to the high *Wolbachia* incidence and diversity observed within this clade.

The high *Wolbachia* incidence found in *Nesobasis* may in part result from the high species richness in this group, but reciprocally, *Wolbachia* may also have contributed to host speciation. Indeed, this symbiont can create or exacerbate reproductive isolation between their hosts when it expresses the CI phenotype. Specifically, the effect on speciation rates will be highest when sister species carry different CI types, that is, mutually incompatible *Wolbachia* strains^16^. Within our data, we observe many different strains in many species, a pattern compatible with a role of *Wolbachia* in reproductive isolation. The likelihood of such a hypothesis will depend on the phenotypic effects that these endosymbionts may have in *Nesobasis*. The observed lack of association between host sex and *Wolbachia* infection status rules out a scenario of male-killing or feminization phenotypes. In addition, for those species reported as having female-biased sex ratios in the field, the prevalence of *Wolbachia* was not higher than that observed in species with 1:1 sex ratios (e.g. *N*. *rufostigma*, *N*. *malcolmi* or *N*. sp. nov. 4, see Table 1), thus excluding parthenogenesis induction. While the high *Wolbachia* frequencies found within *Nesobasis* species are compatible with a CI phenotype^30^, the role of *Wolbachia* in the speciation process still requires direct study of the endosymbiont phenotype in these hosts. While captive rearing and breeding of odonates may be challenging (their dietary requirements as predators in the larval and adult stages can make long-term maintenance difficult, and not all species are amenable to mating in laboratory conditions) the large number of species involved would serve as an abundant resource for experiments.

The high prevalence of *Wolbachia* observed in many of the *Nesobasis* species screened suggests that the efficiency of endosymbiont vertical transmission is high in our sample. At the interspecific level, our ParaFit and PACo global results consistently indicate that the host and symbiont histories are not independent, and indeed the analysis with Jane indicated some likely co-speciation events. However, the observed pattern of *Wolbachia* infection is not explained by vertical transmission alone: the presence of an epidemic *Wolbachia* strain (strain A1) found infecting both *Melanesobasis* and *Nesobasis*, and the fact that some *Nesobasis* species were found harbouring multiple divergent *Wolbachia* strains, constitute evidence for host-switch events. These may involve genuine horizontal transfers. Although *Wolbachia* vectors are still unknown, parasitic mites could be at play as has been recently found in *Drosophila*^31^. Mites are commonly found on odonates^32^, including *Nesobasis* (M. Marinov, personal communication). On the other hand, rare hybridization events between host species could provide a means for horizontal transfer events on a small evolutionary scale, as suggested by the presence of the same or closely related *Wolbachia* in pairs of sister species. *Wolbachia* switches between host lineages through hybridization would generate introgression of the mitochondrial lineages associated with *Wolbachia* but not that of nuclear genes. Hybrid introgression events could thus partly explain the observed conflicts between the nuclear and mitochondrial trees in our data. Further exploration of this hypothesis will require a wider set of nuclear markers than those currently available.

The biogeographical patterns of *Wolbachia* infection among species of *Nesobasis* are interesting to consider in the context of hybridization and speciation. Viti Levu and Vanua Levu both have a large number of *Nesobasis* species with virtually no overlap. Viti Levu is the proposed origin for the radiation of *Nesobasis* within Fiji^23^, with dispersal events between Viti and Vanua Levu having occurred a number of times throughout evolutionary time. Vanua Levu has a significantly higher proportion of species infected with *Wolbachia* (100% in our sample) than Viti Levu (47% of species sampled). While the biogeographical analysis of Beatty *et al*.^23^ suggests that a relatively low number of extinctions are necessary to explain species distributions, it is possible that *Wolbachia*-induced reproductive isolation, paired with dispersal events, explains the higher rate of infection observed in the Vanua Levu species.

The high incidence and diversity of *Wolbachia* in our study system is in line with the hypothesis that this symbiont may spread most efficiently in species-rich groups. Reciprocally, these results open new avenues of research in addressing the question of why there are so many species of *Nesobasis*. Endosymbiont-mediated speciation could have contributed to the species radiation in this remarkable group of island damselflies. Further experiments, including direct assessments of the *Wolbachia*-induced phenotypes, and their effects of gene flow across species, will allow further exploration of this hypothesis.

## Methods

### Sample collection and DNA extraction

Samples of *Nesobasis* and *Melanesobasis* species were collected at several localities in Fiji in 2005 and 2006 (Fig. 1, Suppl. Information Table S1). Adult damselflies were collected using a hand net and preserved in absolute ethanol at 4 °C until DNA extraction. Total genomic DNA was extracted from the subterminal part of the abdomen of each specimen, using the DNeasy tissue kit (Qiagen, Venlo, The Netherlands), following the manufacturer’s protocol for insect tissues.

### *Wolbachia* and host DNA amplification and sequencing

To test for the presence of *Wolbachia* in our damselfly samples, we amplified the *wsp* gene of the endosymbiont, following previously described PCR protocols with primers *wsp*_81F and *wsp*_691R^33^.

*Wolbachia* strains were characterized in a total of 30 individuals (1–2 per species, see Table 1 and Suppl. Information Table S1), using the five MLST housekeeping genes (*gatB*, *coxA*, *hcpA*, *ftsZ* and *fbpA*^34^). Each unique allele from all of the five genes is assigned a number, and hence, each *Wolbachia* strain (ST) is defined by a profile of five allele numbers (allelic profile). Primers specific to *Wolbachia* A and B supergroups for each of the five MLST genes were used for strain typing, following standard PCR protocols^34^ (https://pubmlst.org/*wolbachia*/).

PCR reactions were carried out using the DreamTaq Green PCR Master Mix (ThermoFisher Scientific, Waltham, Massachusetts). Prior to sequencing, unincorporated primers and dNTPs were digested using *Shrimp Alkaline Phosphatase* and *Exonuclease I* (New England Biolabs, Ipswich, Massachusetts, USA). Cleaned PCR products were sequenced in both directions using BigDye v.3.1 chemistry (Applied Biosystems, Foster City, California) and capillary electrophoresis on an ABI3130xl Genetic Analyzer (Applied Biosystems). Chromatograms were visually inspected and assembled with Geneious v. 9.1.7^35^ (https://www.geneious.com).

All consensus sequences were trimmed to the appropriate length for database query, using the templates for each locus found in the MLST database (https://pubmlst.org/*wolbachia*/info/allele_templates.shtml). A BLAST search of each sequence was performed against the *Wolbachia* MLST database^36^ (http://pubmlst.org/*wolbachia*). When a sequence had an exact match in the database, it was assigned the designated allele number otherwise the sequence was submitted to the MLST database for allele number assignment.

To reconstruct the phylogenetic relationships of the host species, fragments of the Arginine Methyltransferase (*PRMT*) nuclear and the mitochondrial Cytochrome Oxidase I (*COI*) genes were amplified using specific primers and annealing temperatures^37–39^. PCR reactions, sequencing and chromatogram inspections were carried out as described above (see Suppl. Information Table S3 for information on primer annealing temperatures). For the *PRMT* locus, ambiguities with similar peak heights were considered to be heterozygous positions and recoded with IUPAC ambiguity codes. Some individuals from the species *Nesobasis brachycerca* (n = 1), *N*. sp. nov. 9 (n = 1), *N*. *leveri* (n = 1) and *N*. *selysi* (n = 2); showed superimposed traces typical of heterozygous indels, and these were resolved using the software Indelligent v.1.2^40^ (http://dmitriev.speciesfile.org/indel.asp). All sequences generated in this study have been submitted to the NCBI GenBank (https://www.ncbi.nlm.nih.gov/genbank/; see Suppl. Information Tables S1 and S2 for accession numbers).

### Genetic data analyses

All sequences were aligned with ClustalW^41^, as implemented in Geneious v. 9.1.7. Phylogenetic relationships among the *Wolbachia* strains found in *Nesobasis* and *Melanesobasis* were reconstructed using ClonalFrame v1.2, a bayesian software that estimates the clonal relationships between the members of a sample, while also estimating the chromosomal position of homologous recombination events that have disrupted the clonal inheritance^42^. The complete dataset used in the ClonalFrame analyses included MLST profile sequences from 101 representative *Wolbachia* strains from A, B, C, F and H supergroups that were downloaded from the MLST database (see Suppl. Information Table S4), together with the MLST strains identifies in the *Nesobasis* and *Melanesobasis* species. Three independent ClonalFrame runs of the dataset were performed, with 500,000 MCMC iterations after 100,000 burn-in iterations and all the rest of the parameters set as default. Convergence of the runs was assessed with the Gelman and Rubin method^43^ as implemented in the ParseCF script written by Barry Hall.

Phylogenetic relationships between the *Nesobasis* and *Melanesobasis* species for both mitochondrial and nuclear DNA were reconstructed under Maximum Likelihood (ML) and Bayesian Inference (BI) frameworks. ML trees were built using the Randomized Axelerated ML algorithms implemented in RaxML-HPC2 v.8.2.8^44^, through the CIPRES web portal (http://www.phylo.org). The analysis was run under the GTR + G model, and bootstrapping was performed under auto majority rule criterion (autoMRE). BI analysis was performed in MrBayes v.3.2.6^45^ implemented in Geneious v. 9.1.7. Searches were run for five million generations, in two independent runs, using the defaults priors and the best fitting nucleotide substitution model for each locus as selected in jModelTest v2.1.10^46,47^. Burn-in samples (first 25% of trees) were discarded, and the remaining were combined to produce a 50% majority rule consensus tree, with bipartition frequencies equal to posterior probabilities values.

### Cophylogenetic analyses

In order to analyze the nature of the interactions between *Wolbachia* and their damselfly hosts, we followed the global-fit cophylogenetic approaches implemented by ParaFit^48^ and PACo^49^; and the event-based method implemented by Jane^50^. For these analyses, the datasets were pruned to contain only those *Wolbachia* infected hosts included in the MLST analysis for which COI sequences were also available.

ParaFit and PACo methods were used to assess the overall congruence between the topologies of the hosts and *Wolbachia*, and to identify those host-*Wolbachia* associations contributing to the detected congruence pattern. Genetic distance matrices (uncorrected p-distances) were obtained using MEGA X^51^ for both the *Wolbachia* concatendated MLST and the host COI datasets. These matrices, together with a matrix representing host-*Wolbachia* associations, were used as input for both analyses (see Suppl. Information Table S6). ParaFit assesses the congruence between phylogenies through a global test of random association between taxa (with a null hypothesis of no relationship), while PACo explicitly tests the dependence between topologies through a Procrustes superimposition of distance matrixes. For the PACo analysis, we tested the hypothesis that both the *Wolbachia* and host phylogenies depend on each other; with the null hypothesis that the *Wolbachia* ordination does not predict the host ordination and vice-versa, such that the host clades are randomly associated to the *Wolbachia* clades. Hypothesis testing was performed by setting the parameters *methods* = “*quasiswap*” and *symmetry* = *T* in the function *PACo*^49^. The relative contribution of individual associations to overall congruence was tested through ParaFitLink1 and ParaFitLink2 tests and assessed through the squared residuals for each association from PACo analysis (non-congruent associations: 95% squared residual confidence interval > median value). All analyses were conducted in R^52^ with the packages *PACo* v0.3.260^53^ and *ape* v5.1^54^. All R scripts and the input data used in these analyses can be found in Suppl. Information Table S6.

In order to assess the contribution of a set of evolutionary events to the observed pattern of association between *Wolbachia* and their hosts, we performed an analysis with the software Jane v4^50^ (https://www.cs.hmc.edu/~hadas/jane/index.html#get). Briefly, Jane attempts to find solutions that minimize the total sum of the event costs through a genetic algorithm. The costs for each evolutionary event were specified as follows: co-speciation = 0 (host and *Wolbachia* speciate simultaneously); duplication = 1 (*Wolbachia* speciates and both species remain on the same host); duplication and host switch = 2 (*Wolbachia* speciates and one of the new species switches onto a different host); loss = 1 (a host speciates and *Wolbachia* is absent in one of the new host species); and failure to diverge = 1 (a host speciates and *Wolbachia* remains on both new host species). *Wolbachia* and host phylogenetic trees were pruned to contain only those individuals for which both MLST and COI sequences were available, using the script *filter_tree*.*py*, provided within Qiime^55^. The pruned trees, together with a mapping of the tips of the parasite tree to tips of the host tree, were used as input for the analysis (see Suppl. Information Table S6). The genetic algorithm was run through 100 generations (G), with a population size (N) = 200; as higher G and N increased computational time without reducing the asymptotic cost of solutions. The robustness of the solution was assessed through a random permutation of tip mappings (G = 100, N = 200, sample size = 100).

## Supplementary information

Supplementary Information File

## References

[1] Werren J, Baldo L, Clark ME (2008). *Wolbachia*: master manipulators of invertebrate biology. Nature Reviews Microbiology..

[2] Zug R, Hammerstein P (2012). Still a host of hosts for *Wolbachia*: analysis of recent data suggests that 40% of terrestrial arthropod species are infected. PLoS One..

[3] Weinert LA, Araujo-Jnr EV, Ahmed MZ, Welch JJ (2015). The incidence of bacterial endosymbionts in terrestrial arthropods. Proc. R. Soc. B..

[4] Sazama EJ, Bosch MJ, Shouldis CS, Ouellette SP, Wesner JS (2017). Incidence of *Wolbachia* in aquatic insects. Ecol Evol..

[5] Sazama EJ, Ouellette SP, Wesner JS (2019). Bacterial endosymbionts are common among, but not necessarily within, insect species. Environ. Entomol..

[6] Zug R, Hammerstein P (2015). Bad guys turned nice? A critical assessment of *Wolbachia* mutualisms in arthropod hosts. Biol Rev..

[7] Duron O, Hurst GDD (2013). Arthropods and inherited bacteria: from counting the symbionts to understanding how symbionts count. BMC Biology..

[8] Frydman HM, Li JM, Robson DN, Wieschaus E (2006). Somatic stem cell niche tropism in *Wolbachia*. Nature..

[9] Correa CC, Ballard JWO (2016). *Wolbachia* associations with insects: winning or losing against a master manipulator. Frontiers in Ecology and Evolution..

[10] Bailly-Bechet M (2017). How long does *Wolbachia* remain on board?. Mol Biol Evol..

[11] Dale C, Moran NA (2006). Molecular interactions between bacterial symbionts and their hosts. Cell..

[12] Raychoudhury R, Baldo L, Oliveira DCSG, Werren JH (2009). Modes of adquisition of *Wolbachia*: horizontal transfer, hybrid introgression and codivergence in the Nasonia species complex. Evolution..

[13] Engelstädter J, Hurst GDD (2006). The dynamics of parasite incidence across host species. Evolutionary Ecology..

[14] Telschow A, Hammerstein P, Werren JH (2005). The effect of *Wolbachia* versus genetic incompatibilities on reinforcement and speciation. Evolution..

[15] Breeuwer JA, Werren JH (1990). Microorganisms associated with chromosome destruction and reproductive isolation between two insect species. Nature..

[16] Bordenstein S, O’Hara P, Werren JH (2001). *Wolbachia*-induced incompatibility precedes other hybrid incompatibilities in Nasonia. Nature..

[17] Donnelly TW (1990). The Fijian genus Nesobasis Part 1: Species of Viti Levu, Ovalau, and Kadavu (Odonata: Coenagrionidae). New Zealand Journal of Zoology..

[18] Jordan S, Simon C, Polhemus D (2003). Molecular systematics and adaptive radiation of Hawaii’s endemic damselfly genus Megalagrion (Odonata: Coenagrionidae). Systematic Biology..

[19] Jordan S, Simon C, Foote D, Englund RA (2005). Phylogeographic patterns of Hawaiian Megalagrion damselflies (Odonata: Coenagrionidae) correlate with Pleistocene island boundaries. Molecular Ecology..

[20] Polhemus DA (1997). Phylogenetic analysis of the Hawaiian damselfly genus Megalagrion (Odonata: Coenagrionidae): Implications for biogeography, ecology, and conservation biology. Pacific Science..

[21] Donnelly TW (1984). Melanesobasis gen. nov., a new genus of Fijian damselflies: A possible link between the platycnemidid Lieftinckia and certain coenagrionids (Zygoptera). Odonatologica..

[22] Van Gossum H (2007). Male rarity and putative sex- role reversal in Fijian damselflies (Odonata). Journal of Tropical Ecology..

[23] Beatty CD (2017). Biogeography and systematics of endemic island damselflies: The Nesobasis and Melanesobasis (Odonata: Coenagrionidae) of Fiji. Ecology & Evolution..

[24] McPeek MA (2007). The macroevolutionary consequences of ecological differences among species. Palaeontology..

[25] McPeek MA (2008). The ecological dynamics of clade diversification and community assembly. The American Naturalist..

[26] Salunkhe RC (2015). Distribution and molecular characterization of *Wolbachia* endosymbionts in Odonata (Insecta) from Central India by multigene approach. Current Science..

[27] Hurst GD, Jiggins FM (2005). Problems with mitochondrial DNA as a marker in population, phylogeographic and phylogenetic studies: the effects of inherited symbionts. Proc. Biol. Sci..

[28] Werren JH, Windsor DM (2000). *Wolbachia* infection frequencies in insects: evidence of a global equilibrium?. Proc Biol Sci..

[29] Hilgenboecker K (2008). How many species are infected with *Wolbachia*? a statistical analysis of current data. FEMS Microbiol. Lett..

[30] Narita S, Shimajiri Y, Nomura M (2009). Strong cytoplasmic incompatibility and high vertical transmission rate can explain the high frequencies of *Wolbachia* infection in Japanese populations of Colias erate poliographus (Lepidoptera: Pieridae). Bulletin of Entomological Research..

[31] Brown AN, Lloyd VK (2015). Evidence for horizontal transfer of *Wolbachia* by a Drosophila mite. Exp Appl Acarol..

[32] Corbet, P. S. Dragonflies: Behaviour and Ecology of Odonata. 829 pp (Harley Books, Colchester, UK, 2004).

[33] Zhou W, Rousset F, O’Neill S (1998). Phylogeny and PCR–based classification of *Wolbachia* strains using wsp gene sequences Proc. R. Soc. Lond. B..

[34] Baldo L (2006). Multilocus sequence typing system for the endosymbiont *Wolbachia* pipientis. Appl Environ Microbiol..

[35] Kearse M (2012). Geneious Basic: an integrated and extendable desktop software platform for the organization and analysis of sequence data. Bioinformatics..

[36] Jolley KA, Chan MS, Maiden MC (2004). J. mlstdbNet-Distributed multi-locus sequence typing (MLST) databases. BMC Bioinformatics..

[37] Dijkstra K-DB, Kalkman VJ, Dow RA, Stokvis FR, Van Tol J (2004). Redefining the damselfly families: a comprehensive molecular phylogeny of Zygoptera (Odonata). Systematic Entomology..

[38] Ferreira S (2014). New EPIC nuclear DNA sequence markers to improve the resolution of phylogeographic studies of coenagrionids and other odonates. International Journal of Odonatology..

[39] Folmer O, Black M, Hoeh W, Lutz R, Vrijenhoek. R (1994). DNA primers for amplification of mitochondrial cytochrome c oxidase subunit I from diverse metazoan invertebrates. Molecular Marine Biology and Biotechnology..

[40] Dmitriev DA, Rakitov RA (2008). Decoding of superimposed traces produced by direct sequencing of heterozygous indels. PLoS Comput. Biol..

[41] Thompson JD, Higgins DG, Gibson TJ (1994). CLUSTAL W: improving the sensitivity of progressive multiple sequence alignment through sequence weighting, position-specific gap penalties and weight matrix choice. Nucleic Acids Res..

[42] Didelot X, Falush D (2007). Inference of bacterial microevolution using multilocus sequence data. Genetics..

[43] Gelman A, Rubin DB (1992). Inference from iterative simulation using multiple sequences. Statist. Sci..

[44] Stamatakis A (2006). RAxML-VI-HPC: maximum likelihood-based phylogenetic analyses with thousands of taxa and mixed models. Bioinformatics..

[45] Ronquist F (2012). MrBayes 3.2: Efficient Bayesian phylogenetic inference and model choice across a large model space. Systematic Biology..

[46] Darriba D, Taboada GL, Doallo R, Posada D (2012). jModelTest 2: more models, new heuristics and parallel computing. Nature Methods..

[47] Guindon S, Gascuel O (2003). A simple, fast and accurate method to estimate large phylogenies by maximum-likelihood. Systematic Biology..

[48] Legendre P, Desdevises Y, Bazin E (2002). A statistical test for host-parasite coevolution. Systematic Biology.

[49] Balbuena JA, Míguez-Lozano R, Blasco-Costa I (2013). PACo: a novel Procrustes application to cophylogenetic analysis. PlosOne..

[50] Conow C, Fielder D, Ovadia Y, Libeskind-Hadas R (2010). Jane: A new tool for the cophylogeny reconstruction problem. Algorithms in Molecular Biology..

[51] Kumar S, Stecher G, Li M, Knyaz C, Tamura K (2018). MEGA X: Molecular Evolutionary Genetics Analysis across computing platforms. Molecular Biology and Evolution..

[52] R Core Team. R: A language and environment for statistical computing. R Foundation for Statistical Computing, Vienna, Austria, https://www.R-project.org/ (2018).

[53] Hutchinson MC, Cagua EF, Balbuena JA, Stouffer DB, Poisot T (2017). paco: implementing Procrustean Approach to Cophylogeny in R. Methods in Ecology and Evolution..

[54] Paradis E, Claude J, Strimmer K (2004). APE: analyses of phylogenetics and evolution in R language. Bioinformatics..

[55] Caporaso JG (2010). QIIME allows analysis of high-throughput community sequencing data. Nature Methods.

