# Supplementary material for: Widespread Wolbachia infection in an insular radiation of damselflies (Odonata, Coenagrionidae)
Source: Sci Rep. 2019 Aug 15;9:11933. doi: 10.1038/s41598-019-47954-3 (PMC6695491; doi:10.1038/s41598-019-47954-3)
Supplement: Supplementary file 1 — Supplementary Information File [file 41598_2019_47954_MOESM1_ESM.pdf]

Supplementary Information File

Widespread *Wolbachia* infection in an insular radiation of damselflies (Odonata, Coenagrionidae).

*M. O. Lorenzo-Carballa[1,2], Y. Torres-Cambas[3], K. Heaton[2], G.D.D. Hurst[2], S. Charlat[4], T. N. Sherratt[5], H. Van Gossum[6], A. Cordero-Rivera[1], C. D. Beatty[7]

[1]*ECOEVO Lab, EE Forestal, Campus Universitario A Xunqueira s/n, 36005, Pontevedra, Spain*

[2]*Institute of Integrative Biology, University of Liverpool, Crown Street, L69 7ZB, Liverpool, United Kingdom*

[3] *Departamento de Biología y Geografía, Facultad de Ciencias Naturales y Exactas, Universidad de Oriente, Patricio Lumumba sn, Santiago de Cuba, Cuba*

[4]*Université de Lyon, Université Lyon 1, CNRS, UMR 5558, Laboratoire de Biométrie et Biologie Evolutive, 16, rue Raphael Dubois, 69622 Villeurbanne, France*

[5]*Department of Biology, Carleton University 1125 Colonel By Drive, Ottawa, ON Canada K1S 5B6*

[6]*Evolutionary Ecology Group, University of Antwerp, Campus Drie Eiken, Universiteitsplein 1 D.D.136 2610 Wilrijk Antwerp, Belgium*

[7]*Department of Ecology & Evolutionary Biology, Cornell University, E149 Corson Hall, 215 Tower Road, Ithaca, NY, 08053, United States of America*

***Corresponding author:** M. Olalla Lorenzo-Carballa; *ECOEVO Lab, EE Forestal, Campus Universitario A Xunqueira s/n, 36005, Pontevedra, Spain;* **email address:** m.o.lorenzo.carballa@gmail.com

Supplementary information Table S1: Specimens of *Nesobasis* and *Melanesobasis* examined in this study. For each individual we list the geographical data of the locality of origin, collection date, sample voucher ID, species, sex, *Wolbachia* infection status (WI), *Wolbachia* strains found in each of the MLST typed individuals (highlighted in bold), and GenBank (https://www.ncbi.nlm.nih.gov/genbank/) accession numbers for the *COI* and *PRMT* sequences. "n.a." indicates that sequences of good quality could not be obtained for that particular specimen/locus.

Island	Site Name	Coordinates	Elevation	Collection Date	Voucher ID	Species	Sex	WI	*Wolbachia* strains	GenBank Accession Nos	
										COI	*PRMT*
**Taveuni**	**Mt. Devo**	**16° 50.46' S; 179° 58.07' W**	**1064 m**	**Jan 2006**	**NE1573**	***Melanesobasis corniculata marginata***	**F**	**+**	**A1**	**n.a.**	**n.a.**
	**Bouma Track Upper Falls**	**16° 49.64' S; 179° 52.79' W**	**82 m**	**Jan 2006**	**NE1596**	***Nesobasis sp. nov. 13***	**M**	**+**	**A2, F4**	**n.a.**	**MH328176**
**Kadavu**	SW Namalata Village	19° 04.19' S; 178° 09.11' E	70 m	Jan 2006	NE1576	*Melanesobasis simmondsi*	F	-		MH348621	MH328045
	**SW Namalata Village**	**19° 04.19' S; 178° 09.11' E**	**70 m**	**Jan 2006**	**NE1575**	***Nesobasis recava***	**M**	**+**	**F2**	**n.a.**	**MH328148**
**Viti Levu**	Waivudawa Creek	18° 05.24' S; 178° 21.11' E	104 m	Jan 2006	NE1583	*Melanesobasis corniculata*	M	-		MH348618	MH328042
	**Ocean Pacific Resort Trail**	**18° 15.27' S; 178° 03.82' E**	**18 m**	**Feb 2006**	**NE1572**	***M. maculosa***	**M**	**+**	**A1**	**MH348620**	**MH328044**
	Colo-i-Suva	18° 03.61' S; 178° 27.69' E	223 m	Jan 2006	NE1589	*M. macleani*	M	-		MH348619	MH328043
	**Abaca Road 3**	**17° 40.11' S; 177° 32.52' E**	**535 m**	**Sept 2005**	**NE0915**	***Nesobasis anguillicolis***	**M**	**+**	**A4, F5**	**MH348628**	**MH328052**
	**Abaca Road 3**	**17° 40.11' S; 177° 32.52' E**	**535 m**	**Sept 20059**	**NE0921**	***N. anguillicolis***	**M**	**+**	**A4, F5**	**MH348629**	**MH328053**
	Abaca Road 3	17° 40.11' S; 177° 32.52' E	535 m	Sept 2005	NE0934	*N. anguillicolis*	M	+		MH348630	MH328054
	Abaca Road 3	17° 40.11' S; 177° 32.52' E	535 m	Sept 2005	NE0943	*N. anguillicolis*	M	+		MH348632	MH328055
	Abaca Road 3	17° 40.11' S; 177° 32.52' E	535 m	Sept 2005	NE1212	*N. anguillicolis*	F	+		MH348633	MH328056
	Abaca Road 3	17° 40.11' S; 177° 32.52' E	535 m	Sept 2005	NE1215	*N. anguillicolis*	F	+		MH348634	MH328057
	Abaca Road 3	17° 40.11' S; 177° 32.52' E	535 m	Sept 2005	NE1224	*N. anguillicolis*	F	+		MH348635	MH328058

Island	Site Name	Coordinates	Elevation	Collection Date	Voucher ID	Species	Sex	WI	*Wolbachia* strains	GenBank Accession Nos	
										COI	***PRMT***
**Viti Levu**	Abaca Road 3	17° 40.11' S; 177° 32.52' E	535 m	Sept 2005	NE1230	*N. anguillicolis*	F	+		MH348636	n.a.
	Abaca Road 3	17° 40.11' S; 177° 32.52' E	535 m	Sept 2005	NE1240	*N. anguillicolis*	F	+		MH348640	MH328059
	Sabeto River	17° 43.81' S; 177° 32.66' E	51 m	Aug 2005	NE1659	*N. anguillicolis*	M	+		MH348638	MH328060
	Vaturu Dam Road 2	17° 46.23' S; 177° 36.58' E	430 m	Aug 2005	NE1660	*N. caerulecaudata*	M	-		n.a.	n.a.
	Namosi Road 6	18° 06.74' S; 178° 11.21' E	265 m	Sept 2005	NE1674	*N. campioni*	F	-		MH348646	MH328068
	Vaturu Dam Road 2	17° 46.23' S; 177° 36.58' E	430 m	Aug 2005	NE1662	*N. flavifrons*	M	-		MH348671	MH328091
	**Abaca Road 3**	**17° 40.11' S; 177° 32.52' E**	**535 m**	**Sept 2005**	**NE0928**	***N. comosa***	**M**	**+**	**A1, B6**	**MH348647**	**MH328069**
	Abaca Road 3	17° 40.11' S; 177° 32.52' E	535 m	Sept 2005	NE0931	*N. comosa*	M	-		MH348648	MH328070
	**Abaca Road 3**	**17° 40.11' S; 177° 32.52' E**	**535 m**	**Sept 2005**	**NE0979**	***N. comosa***	**F**	**+**	**A1**	**MH348649**	**MH328071**
	Abaca Road 3	17° 40.11' S; 177° 32.52' E	535 m	Sept 2005	NE1001	*N. comosa*	F	+		MH348650	MH328072
	Abaca Road 3	17° 40.11' S; 177° 32.52' E	535 m	Sept 2005	NE1099	*N. comosa*	M	+		MH348651	MH328073
	Abaca Road 3	17° 40.11' S; 177° 32.52' E	535 m	Sept 2005	NE1106	*N. comosa*	M	+		n.a.	MH328074
	Abaca Road 3	17° 40.11' S; 177° 32.52' E	535 m	Sept 2005	NE1108	*N. comosa*	F	+		n.a.	n.a.
	Abaca Road 3	17° 40.11' S; 177° 32.52' E	535 m	Sept 2005	NE1111	*N. comosa*	M	+		MH348652	MH328075
	Abaca Road 3	17° 40.11' S; 177° 32.52' E	535 m	Sept 2005	NE1119	*N. comosa*	F	+		MH348653	MH328076
	Abaca Road 3	17° 40.11' S; 177° 32.52' E	535 m	Sept 2005	NE1127	*N. comosa*	F	+		MH348654	MH328077

Island	Site Name	Coordinates	Elevation	Collection Date	Voucher ID	Species	Sex	WI	*Wolbachia* strains	GenBank Accession Nos	
										COI	*PRMT*
**Viti Levu**	Wainikovu	18° 06.39' S; 178° 10.82' E	230 m	Aug 2005	NE1661	*N. comosa*	M	+		MH348655	MH328078
	Wainikovu	18° 06.39' S; 178° 10.82' E	230 m	Aug 2005	NE0012	*N. heteroneura*	F	-		MH348673	MH328093
	**Wainikovu**	**18° 06.39' S; 178° 10.82' E**	**230 m**	**Aug 2005**	**NE0049**	***N. heteroneura***	**F**	**+**	**A1, B7, B6**	**MH348674**	**n.a.**
	**Korowaiwai**	**17° 36.18' S; 177° 56.83' E**	**700 m**	**Aug 2005**	**NE0051**	***N. heteroneura***	**F**	**+**	**A1**	**MH348675**	**n.a.**
	Korowaiwai	17° 36.18' S; 177° 56.83' E	700 m	Aug 2005	NE0089	*N. heteroneura*	F	+		MH348676	MH328094
	Waikubukubu	17°32.84'S 177°56.62' E	210 m	Aug 2005	NE0102	*N. heteroneura*	F	+		MH348677	MH328095
	Waikubukubu	17°32.84'S 177°56.62' E	210 m	Aug 2005	NE0162	*N. heteroneura*	F	+		MH348678	n.a.
	Waikubukubu	17°32.84'S 177°56.62' E	210 m	Sept 2005	NEX22	*N. heteroneura*	F	-		MH348679	MH328096
	Waikubukubu	17°32.84'S 177°56.62' E	210 m	Sept 2005	NEX23	*N. heteroneura*	F	+		MH348680	MH328097
	Waikubukubu	17°32.84'S 177°56.62' E	210 m	Sept 2005	NEX24	*N. heteroneura*	F	+		MH348681	MH328098
	Waikubukubu	17°32.84'S 177°56.62' E	210 m	Sept 2005	NEX25	*N. heteroneura*	F	+		MH348682	MH328099
	Waikubukubu	17°32.84'S 177°56.62' E	210 m	Sept 2005	NEX26	*N. heteroneura*	F	+		MH348683	MH328100
	Waikubukubu	17°32.84'S 177°56.62' E	210 m	Sept 2005	NEX27	*N. heteroneura*	F	+		MH348684	MH328101
	Waikubukubu	17°32.84'S 177°56.62' E	210 m	Sept 2005	NEX28	*N. heteroneura*	F	+		MH348685	MH328102
	Waikubukubu	17°32.84'S 177°56.62' E	210 m	Sept 2005	NEX29	*N. heteroneura*	F	+		MH348686	MH328103
	Waikubukubu	17°32.84'S 177°56.62' E	210 m	Sept 2005	NEX30	*N. heteroneura*	F	+		MH348687	MH328104
	Waikubukubu	17°32.84'S 177°56.62' E	210 m	Sept 2005	NEX31	*N. heteroneura*	F	+		MH348688	MH328105
	Waikubukubu	17°32.84'S 177°56.62' E	210 m	Sept 2005	NEX32	*N. heteroneura*	F	+		MH348689	MH328106
**Viti Levu**	Waikubukubu	17°32.84'S 177°56.62' E	210 m	Sept 2005	NEX33	*N. heteroneura*	F	+		MH348690	MH328107

Island	Site Name	Coordinates	Elevation	Collection Date	Voucher ID	Species	Sex	WI	*Wolbachia* strains	GenBank Accession Nos	
										COI	***PRMT***
	Waikubukubu	17°32.84'S 177°56.62' E	210 m	Sept 2005	NEX34	*N. heteroneura*	F	+		MH348691	MH328108
	Waikubukubu	17°32.84'S 177°56.62' E	210 m	Sept 2005	NEX35	*N. heteroneura*	F	+		MH348692	MH328109
	Waikubukubu	17°32.84'S 177°56.62' E	210 m	Sept 2005	NEX37	*N. heteroneura*	F	+		MH348693	MH328110
	Waikubukubu	17°32.84'S 177°56.62' E	210 m	Sept 2005	NEX54	*N. heteroneura*	M	-		MH348694	MH328111
	Waikubukubu	17°32.84'S 177°56.62' E	210 m	Sept 2005	NEX55	*N. heteroneura*	M	+		MH348695	MH328112
	Waikubukubu	17°32.84'S 177°56.62' E	210 m	Sept 2005	NEX56	*N. heteroneura*	M	+		MH348696	MH328113
	Waikubukubu	17°32.84'S 177°56.62' E	210 m	Sept 2005	NEX57	*N. heteroneura*	M	-		MH348697	MH328114
	Waikubukubu	17°32.84'S 177°56.62' E	210 m	Sept 2005	NEX58	*N. heteroneura*	M	+		MH348698	MH328115
	Waikubukubu	17°32.84'S 177°56.62' E	210 m	Sept 2005	NEX59	*N. heteroneura*	M	+		MH348699	n.a.
	Waikubukubu	17°32.84'S 177°56.62' E	210 m	Sept 2005	NEX60	*N. heteroneura*	M	+		MH348700	MH328116
	Waikubukubu	17°32.84'S 177°56.62' E	210 m	Sept 2005	NEX61	*N. heteroneura*	M	+		MH348701	MH328117
	Waikubukubu	17°32.84'S 177°56.62' E	210 m	Sept 2005	NEX62	*N. heteroneura*	M	+		MH348702	n.a.
	Waikubukubu	17°32.84'S 177°56.62' E	210 m	Sept 2005	NEX63	*N. heteroneura*	M	+		MH348703	MH328118
	Waikubukubu	17°32.84'S 177°56.62' E	210 m	Sept 2005	NEX64	*N. heteroneura*	M	+		MH348704	MH328119
	Waikubukubu	17°32.84'S 177°56.62' E	210 m	Sept 2005	NEX65	*N. heteroneura*	M	+		MH348705	MH328120
	Waikubukubu	17°32.84'S 177°56.62' E	210 m	Sept 2005	NEX66	*N. heteroneura*	M	+		MH348706	MH328121
	Waikubukubu	17°32.84'S 177°56.62' E	210 m	Sept 2005	NEX67	*N. heteroneura*	M	+		MH348707	n.a.
**Viti Levu**	Waikubukubu	17°32.84'S 177°56.62' E	210 m	Sept 2005	NEX68	*N. heteroneura*	M	+		MH348708	n.a.
	Waikubukubu	17°32.84'S 177°56.62' E	210 m	Sept 2005	NEX69	*N. heteroneura*	M	+		MH348709	MH328122

Island	Site Name	Coordinates	Elevation	Collection Date	Voucher ID	Species	Sex	WI	*Wolbachia* strains	GenBank Accession Nos	
										COI	*PRMT*
	**Wainikovu**	**18° 06.39' S; 178° 10.82' E**	**230 m**	**Aug 2005**	**NE0006**	***N. erythrops***	**F**	**+**	**A1, B8**	**MH348657**	**n.a.**
	**Wainikovu**	**18° 06.39' S; 178° 10.82' E**	**230 m**	**Aug 2005**	**NE0043**	***N. erythrops***	**F**	**+**	**A1, B8**	**MH348658**	**MH328080**
	Korowaiwai	17° 36.18' S; 177° 56.83' E	700 m	Aug 2005	NE0065	*N. erythrops*	F	+		MH348659	MH328081
	Korowaiwai	17° 36.18' S; 177° 56.83' E	700 m	Aug 2005	NE0071	*N. erythrops*	F	+		MH348660	n.a.
	Waikubukubu	17°32.84'S 177°56.62' E	210 m	Aug 2005	NE0124	*N. erythrops*	F	+		MH348661	MH328082
	Waikubukubu	17°32.84'S 177°56.62' E	210 m	Aug 2005	NE0126	*N. erythrops*	F	+		MH348662	MH328083
	Abaca Road 3	17° 40.11' S; 177° 32.52' E	535 m	Sept 2005	NE0946	*N. erythrops*	M	+		MH348663	MH328084
	Abaca Road 3	17° 40.11' S; 177° 32.52' E	535 m	Sept 2005	NE0957	*N. erythrops*	F	+		MH348664	MH328085
	Abaca Road 3	17° 40.11' S; 177° 32.52' E	535 m	Sept 2005	NE0966	*N. erythrops*	F	+		MH348665	MH328086
	Abaca Road 3	17° 40.11' S; 177° 32.52' E	535 m	Sept 2005	NE0980	*N. erythrops*	M	+		MH348666	MH328087
	Abaca Road 3	17° 40.11' S; 177° 32.52' E	535 m	Sept 2005	NE0981	*N. erythrops*	M	+		MH348667	MH328088
	Abaca Road 3	17° 40.11' S; 177° 32.52' E	535 m	Sept 2005	NE0983	*N. erythrops*	M	+		MH348668	MH328089
	Abaca Road 3	17° 40.11' S; 177° 32.52' E	535 m	Sept 2005	NE0992	*N. erythrops*	M	+		MH348669	n.a.
	Korowaiwai	17° 36.18' S; 177° 56.83' E	700 m	Aug 2005	NE1656	*N. erythrops*	M	+		MH348670	MH328090
	Waivudawa Creek	18° 5.24' S; 178° 21.11' E	104 m	Jan 2006	NE1585	*N. leveri*	M	-		n.a.	MH328131
	Abaca Road 3	17° 40.11' S; 177° 32.52' E	535 m	Sept 2005	NE0912	*N. longistyla*	M	-		MH348721	MH328132
**Viti Levu**	Abaca Road 3	17° 40.11' S; 177° 32.52' E	535 m	Sept 2005	NE0918	*N. longistyla*	M	-		MH348722	MH328133
	Abaca Road 3	17° 40.11' S; 177° 32.52' E	535 m	Sept 2005	NE0937	*N. longistyla*	M	-		n.a.	MH328134
	Abaca Road 3	17° 40.11' S; 177° 32.52' E	535 m	Sept 2005	NE0998	*N. longistyla*	M	-		MH348723	MH328135

**Island**	**Site Name**	**Coordinates**	**Elevation**	**Collection Date**	**Voucher ID**	**Species**	**Sex**	**WI**	***Wolbachia* strains**	**GenBank Accession Nos**	
										COI	***PRMT***
	Abaca Road 3	17° 40.11' S; 177° 32.52' E	535 m	Sept 2005	NE1010	*N. longistyla*	M	-		n.a.	MH328136
	Vereni Falls	17° 40.27' S; 177° 33.06' E	663 m	Aug 2005	NE1644	*N. longistyla*	M	-		MH348724	MH328137
	Vaturu Dam Road 2	17° 46.23' S; 177° 36.58' E	430 m	Jan 2006	NE1658	*N. longistyla*	M	-		MH348725	MH328138
	Abaca Road 3	17° 40.11' S; 177° 32.52' E	535 m	Sept 2005	NE0916	*N. malcolmi*	F	-		MH348726	MH328139
	Abaca Road 3	17° 40.11' S; 177° 32.52' E	535 m	Sept 2005	NE0919	*N. malcolmi*	F	-		MH348727	MH328140
	Abaca Road 3	17° 40.11' S; 177° 32.52' E	535 m	Sept 2005	NE0923	*N. malcolmi*	F	-		MH348728	MH328141
	Abaca Road 3	17° 40.11' S; 177° 32.52' E	535 m	Sept 2005	NE0926	*N. malcolmi*	F	-		MH348729	MH328142
	**Abaca Road 3**	**17° 40.11' S; 177° 32.52' E**	**535 m**	**Sept 2005**	**NE0927**	***N. malcolmi***	**F**	**+**	**A1, B9**	**MH348730**	**MH328143**
	Abaca Road 3	17° 40.11' S; 177° 32.52' E	535 m	Aug 2005	NE1664	*N. malcolmi*	F	-		MH348731	MH328144
	Vereni Falls	17° 40.27' S; 177° 33.06' E	663 m	Jan 2006	NE1624	*N. monticola*	F	-		MH348732	MH328145
	**Vaqo Creek**	**18° 04.89' S; 178° 26.57' E**	**50 m**	**Aug 2005**	**NE0015**	***N. rufostigma***	**F**	**+**	**A1, A3, F4**	**MH348735**	**MH328150**
	**Vaqo Creek**	**18° 04.89' S; 178° 26.57' E**	**50 m**	**Aug 2005**	**NE0050**	***N. rufostigma***	**F**	**+**	**A2, F4**	**MH348736**	**n.a.**
	Nukunuku	17° 37.11' S, 177° 56.71' E	660 m	Aug 2005	NE0088	*N. rufostigma*	F	-		MH348737	MH328151
	Waikubukubu	17°32.84'S 177°56.62' E	210 m	Aug 2005	NE0111	*N. rufostigma*	M	+		MH348738	MH328152
	Waikubukubu	17°32.84'S 177°56.62' E	210 m	Aug 2005	NE0122	*N. rufostigma*	F	+		MH348739	MH328153
**Viti Levu**	Nukunuku	17° 37.11' S, 177° 56.71' E	660 m	Aug 2005	NE0140	*N. rufostigma*	F	+		MH348740	MH328154
	Waikubukubu	17°32.84'S 177°56.62' E	210 m	Aug 2005	NE0157	*N. rufostigma*	F	+		MH348741	MH328149
	Waikubukubu	17°32.84'S 177°56.62' E	210 m	Aug 2005	NE0187	*N. rufostigma*	M	+		MH348742	MH328155
	Waikubukubu	17°32.84'S 177°56.62' E	210 m	Aug 2005	NE0198	*N. rufostigma*	M	+		MH348743	MH328156

Island	Site Name	Coordinates	Elevation	Collection Date	Voucher ID	Species	Sex	WI	*Wolbachia* strains	GenBank Accession Nos	
										COI	***PRMT***
	Vaqo Creek	18° 04.89' S; 178° 26.57' E	50 m	Aug 2005	NE1654	*N. rufostigma*	F	+		MH348744	MH328157
	Waikubukubu	17°32.84'S 177°56.62' E	210 m	Sept 2005	NE2110	*N. rufostigma*	M	+		MH348745	MH328158
	Waikubukubu	17°32.84'S 177°56.62' E	210 m	Sept 2005	NEX01	*N. rufostigma*	F	+		MH348746	MH328159
	Waikubukubu	17°32.84'S 177°56.62' E	210 m	Sept 2005	NEX02	*N. rufostigma*	F	+		MH348747	MH328160
	Waikubukubu	17°32.84'S 177°56.62' E	210 m	Sept 2005	NEX03	*N. rufostigma*	F	+		MH348748	MH328161
	Waikubukubu	17°32.84'S 177°56.62' E	210 m	Sept 2005	NEX04	*N. rufostigma*	F	+		MH348749	MH328162
	Waikubukubu	17°32.84'S 177°56.62' E	210 m	Sept 2005	NEX05	*N. rufostigma*	F	+		MH348750	MH328163
	Waikubukubu	17°32.84'S 177°56.62' E	210 m	Sept 2005	NEX06	*N. rufostigma*	F	+		MH348751	MH328164
	Waikubukubu	17°32.84'S 177°56.62' E	210 m	Sept 2005	NEX07	*N. rufostigma*	F	+		MH348752	MH328165
	Waikubukubu	17°32.84'S 177°56.62' E	210 m	Sept 2005	NEX11	*N. rufostigma*	F	+		MH348753	MH328166
	Waikubukubu	17°32.84'S 177°56.62' E	210 m	Sept 2005	NEX12	*N. rufostigma*	F	+		MH348754	MH328167
	Waikubukubu	17°32.84'S 177°56.62' E	210 m	Sept 2005	NEX17	*N. rufostigma*	F	+		MH348755	MH328168
	Waikubukubu	17°32.84'S 177°56.62' E	210 m	Sept 2005	NEX18	*N. rufostigma*	F	+		MH348756	MH328169
	Waikubukubu	17°32.84'S 177°56.62' E	210 m	Sept 2005	NEX20	*N. rufostigma*	F	+		MH348757	MH328170
**Viti Levu**	Korowaiwai	17° 36.18' S; 177° 56.83' E	700 m	Aug 2005	NE1657	*N. selysi*	F	-		MH348733	MH328146
	Qualiwana Tributary	17° 36.45' S; 177° 59.34' E	725 m	Aug 2005	NE1570	*N. selysi*	M	-		n.a.	n.a.
	Vaturu Dam Road 2	17° 46.23' S; 177° 36.58' E	430 m	Aug 2005	NER29	*N. selysi*	M	-		MH348758	MH328171
	Vaturu Dam Road 2	17° 46.23' S; 177° 36.58' E	430 m	Aug 2005	NER30	*N. selysi*	M	-		MH348759	MH328172
	Vaturu Dam Road 2	17° 46.23' S; 177° 36.58' E	430 m	Aug 2005	NER31	*N. selysi*	M	-		MH348760	MH328173

Island	Site Name	Coordinates	Elevation	Collection Date	Voucher ID	Species	Sex	WI	*Wolbachia* strains	GenBank Accession Nos	
										COI	*PRMT*
	Vaturu Dam Road 2	17° 46.23' S; 177° 36.58' E	430 m	Aug 2005	NER32	*N. selysi*	M	-		MH348761	MH328174
	Vaturu Dam Road 2	17° 46.23' S; 177° 36.58' E	430 m	Aug 2005	NER33	*N. selysi*	M	-		MH348762	MH328175
	**Abaca Road 2**	**17° 39.99' S; 177° 31.65' E**	**380 m**	**Aug 2005**	**NE1663**	***N. telegastrum***	**M**	**+**	**F8**	**MH348763**	**MH328177**
**Vanua Levu**	**Raviravi Creek**	**16° 36.44' S; 179° 08.87' E**	**90 m**	**Sept 2005**	**NE0613**	***Nesobasis sp. nov. 11***	**M**	**+**	**A1, B3, B5**	**MH348622**	**MH328046**
	Raviravi Creek	16° 36.44' S; 179° 08.87' E	90 m	Sept 2005	NE0616	*N. sp. nov. 11*	M	+		MH348623	MH328049
	**Raviravi Creek**	**16° 36.44' S; 179° 08.87' E**	**90 m**	**Sept 2005**	**NE0638**	***N. sp. nov. 11***	**M**	**+**	**A1, A5, B3, B5**	**MH348624**	**MH328051**
	Raviravi Creek	16° 36.44' S; 179° 08.87' E	90 m	Sept 2005	NE0639	*N. sp. nov. 11*	M	+		MH348625	MH328047
	Raviravi Creek	16° 36.44' S; 179° 08.87' E	90 m	Sept 2005	NE0646	*N. sp. nov. 11*	M	+		MH348626	MH328050
	Saivou Village	16° 36.82' S; 179° 09.11' E	192 m	Sept 2005	NE1669	*N. sp. nov. 11*	M	+		MH348627	MH328048
	**Lomaloma Falls**	**16°37.38'S, 179°10.00' E**	**300 m**	**Sept 2005**	**NE1670**	***N. sp. nov. 3***	**F**	**+**	**F7**	**MH348639**	**MH328061**
	**Raviravi Creek**	**16° 36.44' S; 179° 08.87' E**	**90 m**	**Sept 2005**	**NE0619**	***N. brachycerca***	**M**	**+**	**A1, A3, B1**	**MH348640**	**MH328062**
	Raviravi Creek	16° 36.44' S; 179° 08.87' E	90 m	Sept 2005	NE0629	*N. brachycerca*	M	+		MH348641	MH328063
	Raviravi Creek	16° 36.44' S; 179° 08.87' E	90 m	Sept 2005	NE0635	*N. brachycerca*	M	+		MH348642	MH328064
**Vanua Levu**	Raviravi Creek	16° 36.44' S; 179° 08.87' E	90 m	Sept 2005	NE0669	*N. brachycerca*	M	+		MH348643	MH328065
	Raviravi Creek	16° 36.44' S; 179° 08.87' E	90 m	Sept 2005	NE0677	*N. brachycerca*	M	+		MH348644	MH328066
	**Niuwauvudi Creek**	**16° 38.12' S; 179° 45.18' E**	**230 m**	**Sept 2005**	**NE1666**	***N. brachycerca***	**M**	**+**	**B2, B4, F1, F3**	**MH348645**	**MH328067**
	**Lomaloma Falls**	**16°37.38'S, 179°10.00' E**	**300 m**	**Sept 2005**	**NE1667**	***N. sp. nov. 9***	**F**	**+**	**A7**	**MH348656**	**MH328079**
	**Sauvuqoro Creek**	**16° 38.50' S; 179° 13.50' E**	**250 m**	**Sept 2005**	**NE1672**	***N. sp. nov. 10***	**M**	**+**	**F8**	**MH348672**	**MH328092**
	Raviravi Creek	16° 36.44' S; 179° 08.87' E	90 m	Sept 2005	NE0614	*N. sp. nov. 17*	M	+		MH348710	MH328123

Island	Site Name	Coordinates	Elevation	Collection Date	Voucher ID	Species	Sex	WI	*Wolbachia* strains	GenBank Accession Nos	
										COI	*PRMT*
	**Raviravi Creek**	**16° 36.44' S; 179° 08.87' E**	**90 m**	**Sept 2005**	**NE0615**	***N. sp. nov. 17***	**M**	**+**	**B5**	**MH348711**	**n.a.**
	**Raviravi Creek**	**16° 36.44' S; 179° 08.87' E**	**90 m**	**Sept 2005**	**NE0617**	***N. sp. nov. 17***	**M**	**+**	**B5**	**MH348712**	**n.a.**
	Raviravi Creek	16° 36.44' S; 179° 08.87' E	90 m	Sept 2005	NE0621	*N. sp. nov. 17*	F	+		MH348713	MH328124
	Raviravi Creek	16° 36.44' S; 179° 08.87' E	90 m	Sept 2005	NE0627	*N. sp. nov. 17*	F	+		MH348714	MH328125
	Raviravi Creek	16° 36.44' S; 179° 08.87' E	90 m	Sept 2005	NE0628	*N. sp. nov. 17*	F	+		MH348715	MH328126
	Raviravi Creek	16° 36.44' S; 179° 08.87' E	90 m	Sept 2005	NE0630	*N. sp. nov. 17*	F	+		MH348716	MH328127
	Raviravi Creek	16° 36.44' S; 179° 08.87' E	90 m	Sept 2005	NE0631	*N. sp. nov. 17*	F	+		MH348717	MH328128
	Raviravi Creek	16° 36.44' S; 179° 08.87' E	90 m	Sept 2005	NE0632	*N. sp. nov. 17*	M	+		MH348718	n.a.
	Raviravi Creek	16° 36.44' S; 179° 08.87' E	90 m	Sept 2005	NE0633	*N. sp. nov. 17*	M	+		n.a.	MH328129
	Bagasau Creek	16° 42.90' S; 179° 43.67' E	55 m	Sept 2005	NE1665	*N. sp. nov. 17*	M	+		MH348719	MH328130
	**Lomaloma Falls**	**16°37.38'S, 179°10.00' E**	**300 m**	**Sept 2005**	**NE1668**	***N. sp. nov. 6***	**M**	**+**	**A2, A6, F4**	**MH348734**	**MH328147**
	Lomaloma Falls	16°37.38'S, 179°10.00' E	300 m	Sept 2005	NE0452	*N. sp. nov. 4*	F	-		MH348764	MH328181
**Vanua Levu**	**Lomaloma Falls**	**16°37.38'S, 179°10.00' E**	**300 m**	**Sept 2005**	**NE0455**	***N. sp. nov. 4***	**M**	**+**	**B10**	**MH348765**	**MH328182**
	Lomaloma Falls	16°37.38'S, 179°10.00' E	300 m	Sept 2005	NE0458	*N. sp. nov. 4*	M	-		MH348766	MH328178
	Lomaloma Falls	16°37.38'S, 179°10.00' E	300 m	Sept 2005	NE0460	*N. sp. nov. 4*	F	-		MH348767	MH328183
	Lomaloma Falls	16°37.38'S, 179°10.00' E	300 m	Sept 2005	NE0462	*N. sp. nov. 4*	F	-		MH348768	MH328184
	Lomaloma Falls	16°37.38'S, 179°10.00' E	300 m	Sept 2005	NE0468	*N. sp. nov. 4*	F	-		MH348769	MH328179
	Lomaloma Falls	16°37.38'S, 179°10.00' E	300 m	Sept 2005	NE0469	*N. sp. nov. 4*	F	-		MH348770	MH328185
	Lomaloma Falls	16°37.38'S, 179°10.00' E	300 m	Sept 2005	NE0471	*N. sp. nov. 4*	M	-		MH348771	MH328186

Island	Site Name	Coordinates	Elevation	Collection Date	Voucher ID	Species	Sex	WI	*Wolbachia* strains	GenBank Accession Nos	
										COI	***PRMT***
	Lomaloma Falls	16°37.38'S, 179°10.00' E	300 m	Sept 2005	NE0483	*N. sp. nov. 4*	M	-		MH348772	MH328187
	Lomaloma Falls	16°37.38'S, 179°10.00' E	300 m	Sept 2005	NE0502	*N. sp. nov. 4*	M	-		MH348773	MH328188
	Sauvuqoro Creek	16° 36.44' S; 179° 08.87' E	250 m	Sept 2005	NE1673	*N. sp. nov. 4*	M	-		MH348774	MH328180
	**Lomaloma Falls**	**16°37.38'S, 179°10.00' E**	**300 m**	**Sept 2005**	**NE0457**	***N. sp. nov. 13***	**F**	**+**	**F6**	**n.a.**	**n.a.**
	**Lomaloma Falls**	**16°37.38'S, 179°10.00' E**	**300 m**	**Sept 2005**	**NE0466**	***N. sp. nov. 13***	**F**	**+**	**F6**	**n.a.**	**MH328190**
	Lomaloma Falls	16°37.38'S, 179°10.00' E	300 m	Sept 2005	NE0467	*N. sp. nov. 13*	F	+		n.a.	MH328191
	Lomaloma Falls	16°37.38'S, 179°10.00' E	300 m	Sept 2005	NE0472	*N. sp. nov. 13*	F	+		n.a.	MH328192
	Lomaloma Falls	16°37.38'S, 179°10.00' E	300 m	Sept 2005	NE0474	*N. sp. nov. 13*	M	+		n.a.	MH328193
	Lomaloma Falls	16°37.38'S, 179°10.00' E	300 m	Sept 2005	NE0489	*N. sp. nov. 13*	M	+		n.a.	MH328194
	Lomaloma Falls	16°37.38'S, 179°10.00' E	300 m	Sept 2005	NE0495	*N. sp. nov. 13*	F	+		n.a.	MH328195
	Lomaloma Falls	16°37.38'S, 179°10.00' E	300 m	Sept 2005	NE0498	*N. sp. nov. 13*	M	-		MH348775	MH328196
**Vanua Levu**	Lomaloma Falls	16°37.38'S, 179°10.00' E	300 m	Sept 2005	NE0516	*N. sp. nov. 13*	M	+		n.a.	MH328197
	Lomaloma Falls	16°37.38'S, 179°10.00' E	300 m	Sept 2005	NE0518	*N. sp. nov. 13*	M	+		n.a.	MH328198
	Raviravi Creek	16° 36.44' S; 179° 08.87' E	90 m	Sept 2005	NE1671	*N. sp. nov. 13*	M	+		MH348776	MH328189

Supplementary Information Table S2: GenBank (https://www.ncbi.nlm.nih.gov/genbank/) accession numbers for the *Wolbachia* sequences generated in this study. Listed are: the host species name, host Voucher ID, host sex, the accession number for each of the MLST genes sequenced (gatB, coxA, hcpA, ftsZ and fbpA), the strain ID and the supergroup to which each strain belongs, according to the ClonalFrame analysis (see main text for details). n.a. indicates that a sequence could not be obtained for that particular locus/specimen.

**Species**	**Voucher ID**	**Sex**	**gatB**	**coxA**	**hcpA**	**ftsZ**	**fbpA**	**Strain ID**	**Supergroup**
Melanesobasis corniculata marginata	NE1573	F	MH291003	n.a.	MH290964	MH290919	MH291091	Nesobasis_ST-A1	A
M. maculosa	NE1572	M	MH291004	n.a.	MH290965	MH290920	MH291090	Nesobasis_ST-A1	A
Nesobasis heteroneura	NE0051	F	MH291029	n.a.	MH290990	MH290941	MH291061	Nesobasis_ST-A1	A
N. heteroneura	NE0049	F	MH291030	n.a.	MH290995	MH290943	MH291062	Nesobasis_ST-A1	A
N. erythrops	NE0043	F	MH291026	n.a.	MH290997	MH290945	MH291066	Nesobasis_ST-A1	A
N. erythrops	NE0006	F	MH291025	n.a.	MH291002	MH290949	MH291067	Nesobasis_ST-A1	A
N. malcolmi	NE0927	F	MH291033	n.a.	MH290970	MH290924	MH291056	Nesobasis_ST-A1	A
N. rufostigma	NE0015	F	MH291038	n.a.	MH291000	MH290947	MH291052	Nesobasis_ST-A1	A
N. sp. nov. 11	NE0613	M	MH291005	n.a.	MH290986	MH290938	MH291089	Nesobasis_ST-A1	A
N. sp. nov. 11	NE0638	M	MH291006	n.a.	MH290978	MH290930	MH291088	Nesobasis_ST-A1	A
N. brachycerca	NE0619	M	MH291015	n.a.	MH290981	MH290933	MH291078	Nesobasis_ST-A1	A
N. comosa	NE0928	M	MH291022	n.a.	MH290968	MH290922	MH291071	Nesobasis_ST-A1	A
N. comosa	NE0979	F	MH291023	n.a.	MH290966	MH290921	MH291070	Nesobasis_ST-A1	A
N. rufostigma	NE0050	F	MH291039	n.a.	MH290992	MH290942	MH291051	Nesobasis_ST-A2	A
N. sp. nov. 6	NE1668	M	MH291035	n.a.	MH290954	MH290913	MH291054	Nesobasis_ST-A2	A
N. sp. nov. 13	NE1596	M	MH291041	n.a.	MH290961	MH290917	MH291049	Nesobasis_ST-A2	A
N. rufostigma	NE0015	F	MH291040	n.a.	MH290999	MH290946	MH291050	Nesobasis_ST-A3	A
N. brachycerca	NE0619	M	MH291016	n.a.	MH290980	MH290932	MH291077	Nesobasis_ST-A3	A
N. anguillicolis	NE0921	M	MH291013	n.a.	MH290972	MH290925	MH291082	Nesobasis_ST-A4	A
N. anguillicolis	NE0915	M	MH291012	n.a.	MH290974	MH290926	MH291083	Nesobasis_ST-A4	A

**Species**	**Voucher ID**	**Sex**	**gatB**	**coxA**	**hcpA**	**ftsZ**	**fbpA**	**Strain ID**	**Supergroup**
N. sp. nov. 11	NE0638	M	MH291007	n.a.	MH290977	MH290929	n.a.	Nesobasis_ST-A5	A
N. sp. nov. 6	NE1668	M	MH291036	n.a.	MH290953	MH290914	n.a.	Nesobasis_ST-A6	A
N. sp. nov. 9	NE1667	F	MH291024	MH290887	MH290955	n.a.	MH291068	Nesobasis_ST-A7	A
N. brachycerca	NE0619	M	MH291017	MH290899	MH290979	MH290931	MH291076	Nesobasis_ST-B1	B
N. brachycerca	NE1666	M	MH291018	n.a.	MH290957	MH290916	MH291075	Nesobasis_ST-B2	B
N. sp. nov. 11	NE0613	M	MH291008	MH290903	MH290985	MH290937	MH291087	Nesobasis_ST-B3	B
N. sp. nov. 11	NE0638	M	MH291009	MH290898	MH290976	MH290928	MH291086	Nesobasis_ST-B3	B
N. brachycerca	NE1666	M	MH291019	n.a.	MH290956	MH290915	MH291074	Nesobasis_ST-B4	B
N. sp. nov. 11	NE0613	M	MH291010	MH290902	MH290984	MH290936	MH291085	Nesobasis_ST-B5	B
N. latistyla	NE0615	M	MH291031	MH290901	MH290983	MH290935	MH291058	Nesobasis_ST-B5	B
N. latistyla	NE0617	M	MH291032	MH290900	MH290982	MH290934	MH291057	Nesobasis_ST-B5	B
N. sp. nov. 11	NE0638	M	MH291011	MH290897	MH290975	MH290927	MH291084	Nesobasis_ST-B5	B
N. comosa	NE0928	M	n.a.	MH290893	MH290967	n.a.	MH291069	Nesobasis_ST-B6	B
N. heteroneura	NE0049	F	n.a.	MH290909	MH290994	n.a.	MH291060	Nesobasis_ST-B6	B
N. heteroneura	NE0049	F	n.a.	MH290908	MH290993	n.a.	MH291059	Nesobasis_ST-B7	B
N. erythrops	NE0006	F	MH291027	MH290912	MH291001	MH290948	MH291065	Nesobasis_ST-B8	B
N. erythrops	NE0043	F	MH291028	MH290910	MH290996	MH290944	MH291064	Nesobasis_ST-B8	B
N. malcolmi	NE0927	F	MH291034	MH290894	MH290969	MH290923	MH291055	Nesobasis_ST-B9	B
N. sp. nov. 4	NE0455	M	MH291042	MH290906	MH290989	n.a.	MH291047	Nesobasis_ST-B10	B
N. brachycerca	NE1666	M	MH291020	MH290889	MH290959	n.a.	MH291073	Nesobasis_ST-F1	F
N. recava	NE1575	M	MH291037	MH290892	MH290963	MH290918	MH291053	Nesobasis_ST-F2	F
N. brachycerca	NE1666	M	MH291021	MH290888	MH290958	n.a.	MH291072	Nesobasis_ST-F3	F

Species	Voucher ID	Sex	gatB	coxA	hcpA	ftsZ	fbpA	Strain ID	Supergroup
N. sp. nov. 13	NE1596	M	n.a.	MH290891	MH290962	n.a.	n.a.	Nesobasis_ST-F4	F
N. sp. nov. 6	NE1668	M	n.a.	MH290886	MH290952	n.a.	n.a.	Nesobasis_ST-F4	F
N. rufostigma	NE0050	F	n.a.	MH290907	MH290991	n.a.	n.a.	Nesobasis_ST-F4	F
N. rufostigma	NE0015	F	n.a.	MH290911	MH290998	n.a.	n.a.	Nesobasis_ST-F4	F
N. anguillicolis	NE0915	M	n.a.	MH290896	MH290973	n.a.	MH291081	Nesobasis_ST-F5	F
N. anguillicolis	NE0921	M	n.a.	MH290895	MH290971	n.a.	MH291080	Nesobasis_ST-F5	F
N. sp. nov. 13	NE0457	F	MH291043	MH290905	MH290988	MH290940	MH291046	Nesobasis_ST-F6	F
N. sp. nov. 13	NE0466	F	MH291044	MH290904	MH290987	MH290939	MH291045	Nesobasis_ST-F6	F
N. sp. nov. 3	NE1670	F	MH291014	MH290885	MH290951	n.a.	MH291079	Nesobasis_ST-F7	F
N. sp. nov. 10	NE1672	M	n.a.	MH290884	MH290950	n.a.	MH291063	Nesobasis_ST-F8	F
N. telegastrum	NE1663	M	n.a.	MH290890	MH290960	n.a.	MH291048	Nesobasis_ST-F8	F

Supplementary Information Table S3: Primer sequences, primer combinations and PCR conditions used to amplify *Nesobasis* and *Melanesobasis* nuclear and mitochondrial DNA in this study.

Primer name	Target locus	Direction	Sequence	Annealing temperature (Ta)	Reference
LCO 1490	COI	F	GGTCAACAAATCATAAAGATATTGG	50 ºC	Folmer et al. (1994)
HCO 2198	COI	R	TCAGGGTGACCAAAAAATCA	50 ºC	Folmer et al. (1994)
ODO_LCO1490d	COI	F	TTTCTACWAACCAYAAAGATATTGG	50 ºC	Dijkstra et al. (2014)
ODO_HCO2198d	COI	R	TAAACTTCWGGRTGTCCAAARAATCA	50 ºC	Dijkstra et al. (2014)
ARG_F2	PRMT	F	TGC CGC CAA GGC TGG AGC ATC	52 ºC	Ferreira et al. (2014)
ARG_F3	PRMT	F	CCG GAA CTC TAT GTA CCA CAA C	52 ºC	Ferreira et al. (2014)
ARG_F4	PRMT	F	TCG ACT CGT ATG CGC ATT TCG G	52 ºC	Ferreira et al. (2014)
ARG_R3	PRMT	R	TGC CAC CTT CCT AAT AGA GCT C	52 ºC	Ferreira et al. (2014)

The PRMT primers were used in the following combinations depending on the species:

- **Combination 1 [ARG_R3/F2]:** *Melanesobasis simmondsii, M. maculosa, Nesobasis flavifrons, N. comosa, N. leveri, N. longistyla, N. malcolmi, N. selysi, N. sp. nov. 10, N. sp. nov. 17.*

- **Combination 2 [ARG_R3/F3]:** *Melanesobasis corniculata, M. macleani, Nesobasis sp nov. 13, N. recava, N. anguillicolis, N. campioni, N. erythrops, N. monticola, N. rufostigma, N. telegastrum, N. brachycerca, N. sp. nov. 9, N. sp. nov. 6.*

- **Combination 3 [ARG_R3/F4]:** *Nesobasis sp. nov. 13, N. sp. nov. 4, N. sp. nov. 3, N. sp. nov. 11, N. heteroneura.*

References:

Dijkstra K-DB, Kalkman VJ, Dow RA, Stokvis FR and Van Tol J (2014) Redefining the damselfly families: a comprehensive molecular phylogeny of Zygoptera (Odonata). *Systematic Entomology*, 39 (1), 68-96.

Ferreira S, Lorenzo-Carballa MO, Torres-Cambas Y, Cordero-Rivera A, Thompson DJ and Watts PC (2014) New EPIC nuclear DNA sequence markers to improve the resolution of phylogeographic studies of coenagrionids and other odonates. *International Journal of Odonatology*, 17 (1-2), 135-147.

Folmer O, Black M, Hoeh W, Lutz R and Vrijenhoek R (1994) DNA primers for amplification of mitochondrial cytochrome c oxidase subunit I from diverse metazoan invertebrates. *Molecular Marine Biology and Biotechnology*, 3(5), 294-299.

Supplementary Information Table S4: *Wolbachia* strains downloaded from the MLST database and used in the ClonalFrame analysis. Listed are the strain ID, strain name, supergroup to which each strain belongs, and host taxonomic information. *n.a.* indicates that no data were available in the database, and in some cases, no information was available for all the fields, but the sequences were downloaded anyway as they appeared as closely related to the *Wolbachia* strains identified in our study.

Strain id	Strain Name	Supergroup	Host Class	Host Order	Host Family	Host species
169	Anae_A_TD23	A	Arachnida	Araneae	Agelenidae	*Agelenopsis naevia*
613	Ocac_A_wVdO	A	Insecta	Coleoptera	Chrysomelidae	*Oreina cacaliae*
12	Aalb_A	A	Insecta	Diptera	Culicidae	*Aedes albopictus*
41	A_PanBCI_Calyp16	A	Insecta	Diptera	n.a.	*n.a.*
1	Dmel_A_wMel	A	Insecta	Diptera	Drosophilidae	*Drosophila melanogaster*
344	Gmormor_A_12.3A	A	Insecta	Diptera	Glossinidae	*Glossina morsitans morsitans*
425	FUM_32_A	A	Insecta	Diptera	Tephritidae	*Carpomya vesuviana*
497	Amet_A	A	Insecta	Diptera	Culicidae	*Aedes metallicus*
607	Dsuz_A_wSuz	A	Insecta	Diptera	Drosophilidae	*Drosophila suzukii*
4	Cpen_A	A	Insecta	Hymenoptera	Formicidae	*Camponotus pennsylvanicus*
15	Muni_A	A	Insecta	Hymenoptera	Pteromalidae	*Muscidifurax uniraptor*
17	Ngir_A_16.2	A	Insecta	Hymenoptera	Pteromalidae	*Nasonia giraulti*
2	Sinv_A	A	Insecta	Hymenoptera	Formicidae	*Solenopsis invicta*
106	Azt_A	A	Insecta	Hymenoptera	Formicidae	*Azteca sp.*
129	Dele_A	A	Insecta	Hymenoptera	Formicidae	*Dorymyrmex elegans*
142	Epa_A	A	Insecta	Hymenoptera	Pompilidae	*Evagetes parvus*
148	Mchi_A	A	Insecta	Hymenoptera	Formicidae	*Monomorium chinese*
134	Oclar_A	A	Insecta	Hymenoptera	Formicidae	*Odontomachus clarus*
141	Phe_A_423	A	Insecta	Hymenoptera	Formicidae	*Pheidole*
122	Rmet_A	A	Insecta	Hymenoptera	Formicidae	*Rhytidoponera metallica*
400	Ach_A_wCh2	A	Insecta	Hymenoptera	Bracomdae	*Apanteles chilonis*
451	Aart_A	A	Insecta	Lepidoptera	Lycaenidae	*Aricia artaxerxes*
1644	Mari-A-TGim	A	Insecta	Lepidoptera	Lycaenidae	*Maculinea arion ('summer')*
26	Dsim_B_wMa	B	Insecta	Diptera	Drosophilidae	*Drosophila simulans*
21	Psia_B_00189	B	Insecta	Diptera	Calliphoridae	*Protocalliphora sialia*
70	wCer5_B	B	Insecta	Diptera	Tephritidae	*Rhagoletis cerasi*
76	B_NY_Calyp101204	B	Insecta	Diptera	n.a.	*n.a.*
153	B_NY_Chloro150737a	B	Insecta	Diptera	Chloropidae	*n.a.*
93	B_SFChl1	B	Insecta	Diptera	Chloropidae	*n.a.*
94	B_SFChl2	B	Insecta	Diptera	Chloropidae	*n.a.*
87	Dinn_B	B	Insecta	Diptera	Drosophilidae	*Drosophila innubila*
1630	Svar_B	B	Insecta	Coleoptera	Curculionidae	*Steriphus variabilis*
19	Calt_B	B	Insecta	Coleoptera	Chrysomelidae	*Chelymorpha alternans*
20	Tcon_B_BhAvill AK	B	Insecta	Coleoptera	Tenebrionidae	*Tribolium confusum*
284	Lory_B_wOry1	B	Insecta	Coleoptera	Curculionidae	*Lissorhoptrus oryzophilus*
267	Dcit_B_wDc01	B	Insecta	Hemiptera	Psyllidae	*Diaphorina citri*
320	wBtab	B	Insecta	Hemiptera	Aleyrodidae	*Bemisia tabaci*
314	wMfas1	B	Insecta	Hemiptera	Cicadellidae	*Macrosteles fascifrons*
95	B_tab	B	Insecta	Homoptera	Aleyrodidae	*Bemisia tabaci*
266	Ohei_F_TLR	B	Insecta	Isoptera	Rhinotermitidae	*Coptotermes heimi*

Strain id	Strain Name	Supergroup	Host Class	Host Order	Host Family	Host species
1684	Hhor_B_wHho	B	Insecta	Hymenoptera	Ichneumonidae	*Hyposoter horticola*
1687	Hhor_B_wHho	B	Insecta	Hymenoptera	Ichneumonidae	*Hyposoter horticola*
34	Nvit_B_4.9	B	Insecta	Hymenoptera	Pteromalidae	*Nasonia vitripennis*
92	B_Polybia10	B	Insecta	Hymenoptera	Vespidae	*Polybia sp.*
22	Aenc_B_Ugardan	B	Insecta	Lepidoptera	Nymphalidae	*Acraea encedon*
40	Hbol_B_wBol1	B	Insecta	Lepidoptera	Nymphalidae	*Hypolimnas bolina*
39	Lida_B	B	Insecta	Lepidoptera	Lycaenidae	*Lycaeides idas*
32	Osca_B	B	Insecta	Lepidoptera	Crambidae	*Ostrinia scapulalis*
193	Cela_B	B	Insecta	Lepidoptera	Pieridae	*Colias elate poliographus*
201	Eman_B_Fem	B	Insecta	Lepidoptera	Pieridae	*Eurema mandarina*
99	Hony_B	B	Insecta	Lepidoptera	Lycaenidae	*Horaga onyx*
100	Sviv_B	B	Insecta	Lepidoptera	Lycaenidae	*Surendra vivarna*
211	Ccal_B_wCal	B	Insecta	Lepidoptera	Lycaenidae	*Caleta caleta*
214	Cpom_B_wPom	B	Insecta	Lepidoptera	Pieridae	*Catopsilia pomona*
215	Cros_B_wRos	B	Insecta	Lepidoptera	Lycaenidae	*Castalius rosimon*
217	Dchr_B_wChr	B	Insecta	Lepidoptera	Nymphalidae	*Danaus chysippus*
219	Ehec_B_wHec	B	Insecta	Lepidoptera	Pieridae	*Eurema hecabe*
224	Elae_B_wLae	B	Insecta	Lepidoptera	Pieridae	*Eurema laeta*
239	Pdem_B_wDem	B	Insecta	Lepidoptera	Papilionidae	*Papilio demoleus*
450	Apun_B	B	Insecta	Lepidoptera	Pterophoridae	*Amblyptilia punctidactyla*
452	Cpam_B	B	Insecta	Lepidoptera	Nymphalidae	*Coenonympha pamphilus*
474	Tfis_B	B	Insecta	Lepidoptera	Lycaenidae	*Tongeia fischeri*
1650	Malc-B-Hku1	B	Insecta	Lepidoptera	Lycaenidae	*Maculinea alcon ('cruciata')*
25	Ttai_B	B	Insecta	Orthoptera	Gryllidae	*Teleogryllus taiwanemma*
388	Tpal_B_Odo28	B	Insecta	Odonata	Libellulidae	*Trithemis pallidinervis*
60	Oamm_F_100798	F	Arachnida	Scorpiones	Scorpionidae	*Opistophthalmus ammopus*
265	Ohei_F_Termite3	F	Insecta	Isoptera	Rhinotermitidae	*Coptotermes heimi*
36	Clec_F	F	Insecta	Heteroptera	Cimicidae	*Cimex lectularius*
254	Ohor_F_T1	F	Insecta	Isoptera	Termitidae	*Odontotermes horni*
396	Arr_F	F	Insecta	Actinedida	Arrenuridae	*Parasite mite*
359	Agut_F_Odo2	F	Insecta	Odonata	Aeshnidae	*Anax guttatus*
358	Dtri_F_Odo1	F	Insecta	Odonata	Libellulidae	*Diplacodes trivialis*
372	Apal_F_Odo13	F	Insecta	Odonata	Coenagrionidae	*Aciagrion pallidum*
371	Apan_F_Odo12	F	Insecta	Odonata	Libellulidae	*Acisoma panorpoides panorpoides*
379	Bcon_F_Odo20	F	Insecta	Odonata	Libellulidae	*Brachythemis contaminata*
360	Bcon_F_Odo3	F	Insecta	Odonata	Libellulidae	*Brachythemis contaminata*
386	Ccor_F_Odo26	F	Insecta	Odonata	Coenagrionidae	*Ceriagrion coromandelianum*
384	Cser_F_Odo24	F	Insecta	Odonata	Libellulidae	*Crocothemis servilia*
394	Cser_F_Odo34	F	Insecta	Odonata	Libellulidae	*Crocothemis servilia*
377	Evit_F_Odo18	F	Insecta	Odonata	Macromiidae	*Epophthalmia vittata*
380	Irap_F_Odo21	F	Insecta	Odonata	Gomphidae	*Ictinogomphus rapax*
381	Irap_F_Odo21	F	Insecta	Odonata	Gomphidae	*Ictinogomphus rapax*
367	Isen_F_Odo8	F	Insecta	Odonata	Coenagrionidae	*Ischnura senegalensis*
370	Ntul_F_Odo11	F	Insecta	Odonata	Libellulidae	*Neurothemis tullia*
375	Pfla_F_Odo16	F	Insecta	Odonata	Libellulidae	*Pantala flavescens*
361	Pfla_F_Odo4	F	Insecta	Odonata	Libellulidae	*Pantala flavescens*

Strain id	Strain Name	Supergroup	Host Class	Host Order	Host Family	Host species
393	Tpal_F_Odo33	F	Insecta	Odonata	Libellulidae	*Trithemis pallidinervis*
207	Zang_H	H	Insecta	Isoptera	Hodotermitidae	*Zootermes angusticollis*
505	Ocer_C_OC1	C	Secernentea	Spirurida	Onchocercidae	*Onchocerca cervipedis*
492	Osyl	n.a.	Insecta	Lepidoptera	Hesperiidae	*Ochlodes sylvanus*
479	Cpln	n.a.	Insecta	Lepidoptera	Hesperiidae	*Carterocephalus*
202	n.a.	n.a.	n.a.	n.a.	n.a.	*n.a.*
203	n.a.	n.a.	n.a.	n.a.	n.a.	*n.a.*
204	n.a.	n.a.	n.a.	n.a.	n.a.	*n.a.*
489	n.a.	n.a.	n.a.	n.a.	n.a.	*n.a.*
638	n.a.	n.a.	n.a.	n.a.	n.a.	*n.a.*
1614	n.a.	n.a.	n.a.	n.a.	n.a.	*n.a.*
1703	n.a.	n.a.	n.a.	n.a.	n.a.	*n.a.*
1775	n.a.	n.a.	n.a.	n.a.	n.a.	*n.a.*
1784	n.a.	n.a.	n.a.	n.a.	n.a.	*n.a.*
1801	n.a.	n.a.	n.a.	n.a.	n.a.	*n.a.*

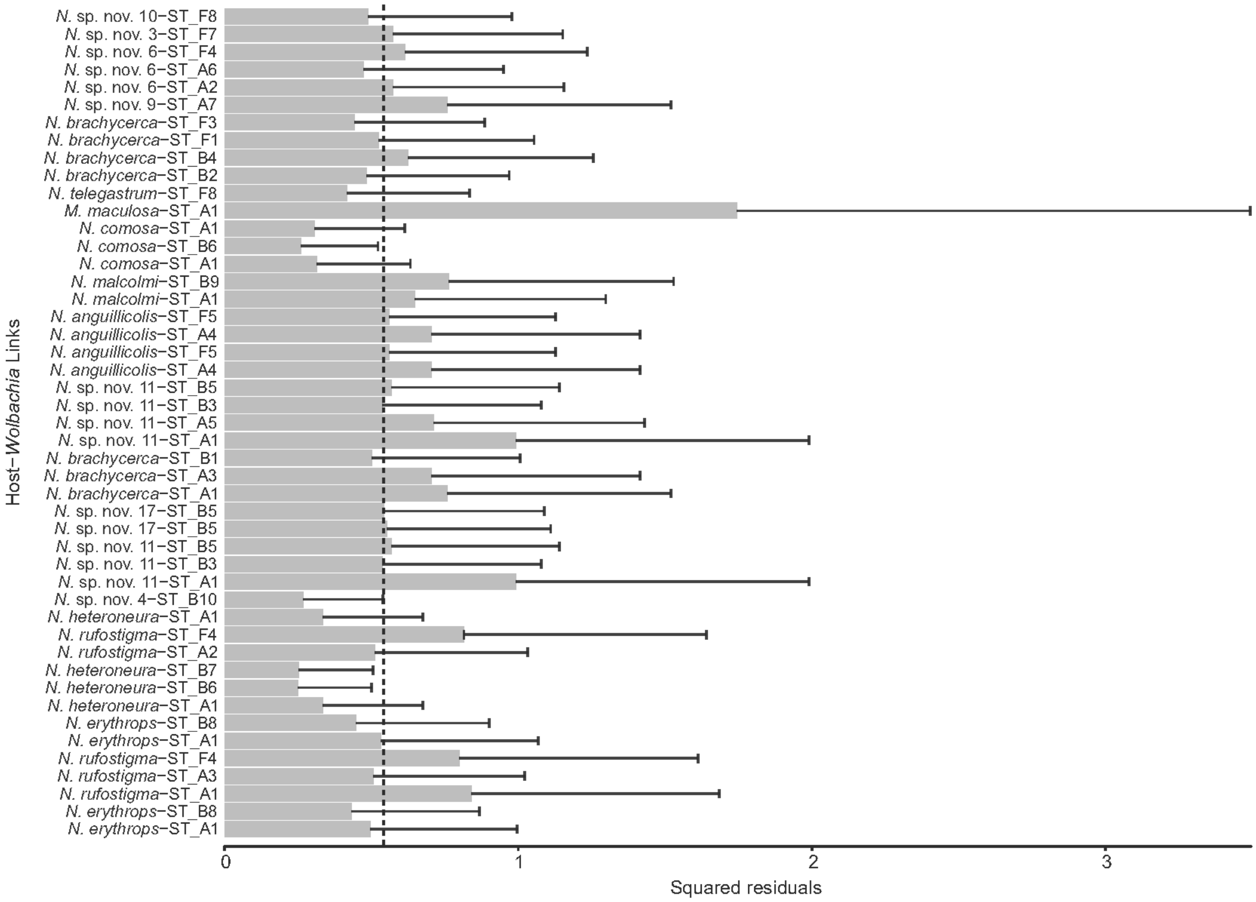

Supplementary Information Figure S1: Contribution of individual associations to the overall topological congruence between phylogenies of *Wolbachia* strands and their *Nesobasis* and *Melanesobasis* damselfly hosts. Gray bars and black bars represent squared residual values and 95% confidence interval values respectively for each association from a Procrustean Approach to Cophylogeny (PACo) analysis. The stippled line indicates the median squared residual value. Associations whose 95% confidence interval values were below the median squared residual represent probable congruent associations.

Supplementary Information Table S5: Results of the ParaFitLink1 and ParaFitLink2 tests for the contribution of individual associations between *Wolbachia* strands and their *Nesobasis* and *Melanesobasis* damselfly hosts to the overall cophylogenetic pattern. For species names corresponding to individual host IDs, see Suppl. Information Table S1.

**Associations**		**ParaFitLink1 test**		**ParaFitLink2 test**	
**Host**	***Wolbachia***	**F1.stat**	**p**	**F2.stat**	**p**
NE0613	ST_B3	0.00238445	0.00799201	0.01545745	0.00799201
NE0613	ST_B5	0.002706842	0.00999001	0.01754739	0.00899101
NE0613	ST_A1	-0.000953329	0.86813187	-0.00618006	0.89210789
NE0638	ST_B3	0.00237873	0.01298701	0.01542037	0.01098901
NE0638	ST_B5	0.00270288	0.00699301	0.01752171	0.00599401
NE0638	ST_A5	0.000332629	0.64135864	0.00215631	0.62237762
NE0638	ST_A1	-0.000944015	0.86813187	-0.00611968	0.89310689
NE0455	ST_B10	0.00106267	0.18081918	0.00688887	0.14485514
NE0915	ST_A4	0.001298678	0.13986014	0.00841882	0.12187812
NE0915	ST_F5	0.001874941	0.02997003	0.0121545	0.02197802
NE0921	ST_A4	0.001298678	0.15484515	0.00841882	0.13786214
NE0921	ST_F5	0.001874941	0.03196803	0.0121545	0.02297702
NE1670	ST_F7	0.00106455	0.16383616	0.00690106	0.13686314
NE0050	ST_A2	0.000833584	0.31668332	0.0054038	0.27472527
NE0050	ST_F4	0.000629591	0.36463536	0.00408139	0.33566434
NE0015	ST_A3	0.000578348	0.45954046	0.0037492	0.41758242
NE0015	ST_A1	-0.001576255	0.95704296	-0.01021824	0.96503497
NE0015	ST_F4	0.000643488	0.37362637	0.00417148	0.33966034
NE1666	ST_B2	0.000529945	0.42257742	0.00343543	0.38561439
NE1666	ST_B4	0.000243109	0.68531469	0.00157598	0.67132867
NE1666	ST_F3	0.000801282	0.28071928	0.00519439	0.23276723
NE1666	ST_F1	0.000803827	0.32067932	0.00521089	0.29270729
NE0619	ST_B1	0.000224591	0.64635365	0.00145593	0.63336663
NE0619	ST_A3	-0.000156271	0.83516484	-0.00101305	0.83716284
NE0619	ST_A1	-0.00132479	0.93406593	-0.00858809	0.95404595
NE0006	ST_B8	0.001353076	0.11188811	0.00877146	0.08891109
NE0006	ST_A1	8.86E-05	0.47152847	0.00057422	0.46653347
NE0043	ST_B8	0.001411156	0.09190809	0.00914797	0.07292707
NE0043	ST_A1	3.93E-05	0.4985015	0.00025451	0.4975025
NE1663	ST_F8	0.000588813	0.41158841	0.00381704	0.38561439
NE0617	ST_B5	0.001558968	0.07492507	0.01010617	0.05694306
NE0615	ST_B5	0.001693112	0.06293706	0.01097578	0.04895105
NE1672	ST_F8	0.000996979	0.20779221	0.00646302	0.16883117
NE1688	ST_A2	0.000442677	0.55844156	0.0028697	0.54345654
NE1688	ST_A6	0.000362515	0.6003996	0.00235004	0.58541459
NE1688	ST_F4	0.000314776	0.51948052	0.00204057	0.4985015
NE0928	ST_B6	0.001891783	0.02597403	0.01226369	0.02097902

Associations		ParaFitLink1 test		ParaFitLink2 test	
**Host**	***Wolbachia***	**F1.stat**	**p**	**F2.stat**	**p**
NE0928	ST_A1	0.002180987	0.01598402	0.01413848	0.01298701
NE0049	ST_B7	0.001462636	0.05694306	0.00948169	0.04295704
NE0049	ST_B6	0.001828506	0.02197802	0.01185348	0.01298701
NE0049	ST_A1	0.002089417	0.01298701	0.01354487	0.00799201v
NE0051	ST_A1	0.002083557	0.01398601	0.01350688	0.00899101
NE0979	ST_A1	0.002162668	0.01298701	0.01401973	0.00599401
NE1667	ST_A7	0.001675866	0.01198801	0.01086398	0.00599401
NE0927	ST_B9	0.000759707	0.25374625	0.00492488	0.20779221
NE0927	ST_A1	0.001068622	0.12487512	0.00692746	0.11288711
NE1572	ST_A1	0.000520978	0.2987013	0.00337729	0.28771229

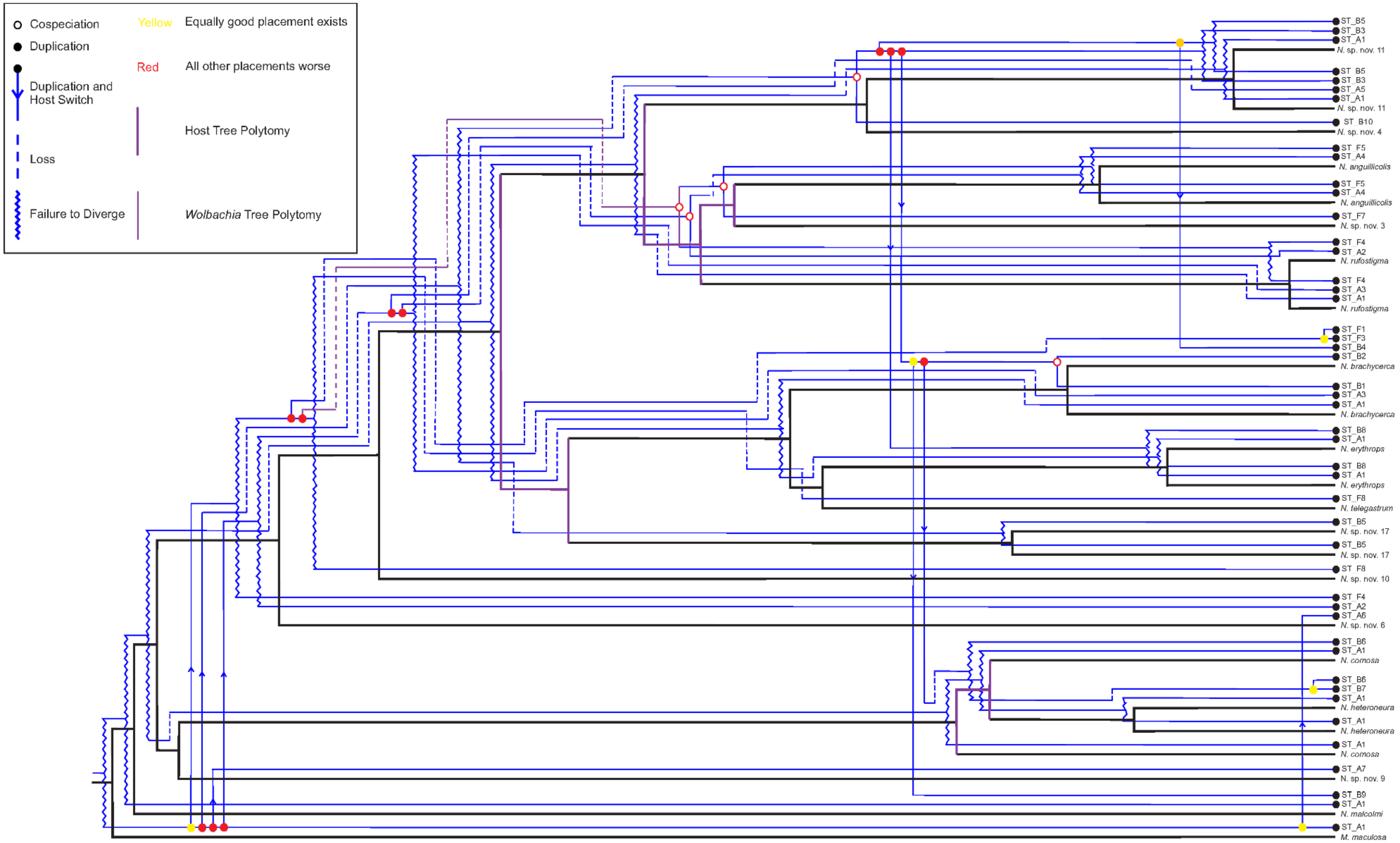

Supplementary Information Figure S2: One of 12 possible solutions found by the program Jane to reconstruct the cophylogentic relation between *Wolbachia* strains and their *Nesobasis* and *Melanesobasis* damselfly hosts. Black and blue lines represent the host and *Wolbachia* phylogenies respectively. Multifurcations in both phylogenies were interpreted as soft polytomies and resolved by the Jane algorithm. The evolutionary events were cospeciation, duplication, duplication and host switch, loss, failure to diverge. A yellow node indicates that there is another location of equal cost, among the possible solutions, where the *Wolbachia* node and its descendent may be mapped. A red node means that all other possible placements have the same cost.

Supplementary Information S6: Input data used in the cophylogenetic analyses carried out for study.

S6-1: Host COI Tree:

(((((((((NE0613:0.000978,NE0638:0.001006):0.081167,NE0455:0.080094):0.012257,
(NE0915:0.001011,NE0921:0.001006):0.078782,NE1670:0.077919,
(NE0050:0.000983,NE0015:0.00483):0.106499):0.028659,
((NE1666:0.002408,NE0619:0.003166):0.078221,
((NE0006:0.001045,NE0043:0.002577):0.059155,NE1663:0.079338):0.009521):0.02149
7,
(NE0617:0.001022,NE0615:0.004822):0.082516):0.037045,NE1672:0.057495):0.018029
,NE1668:0.055614):0.011657,((NE0928:0.001079,
(NE0049:0.001064,NE0051:0.001021):0.013293,NE0979:0.003774):0.036231,NE1667:0.
087753):0.024069):0.023326,NE0927:0.046626):0.0010635,NE1572:0.2423405);

S6-2: Host COI genetic distance matrix:

	NE0613	NE0638	NE0455	NE0915	NE0921	NE1670	NE0050	NE0015	NE1666	NE0619	NE0006	NE0043	NE1663	NE0617	NE0615	NE1672	NE1668	NE0928	NE0049	NE0051	NE0979	NE1667	NE0927	NE1572
NE0613	0	0.0000	0.1083	0.1270	0.1270	0.1145	0.1279	0.1281	0.1369	0.1333	0.1244	0.1261	0.1181	0.1303	0.1303	0.1366	0.1330	0.1340	0.1323	0.1323	0.1340	0.1409	0.1477	0.1966
NE0638	0.0000	0	0.1083	0.1270	0.1270	0.1145	0.1279	0.1281	0.1369	0.1333	0.1244	0.1261	0.1181	0.1303	0.1303	0.1366	0.1330	0.1340	0.1323	0.1323	0.1340	0.1409	0.1477	0.1966
NE0455	0.1083	0.1083	0	0.1052	0.1052	0.1136	0.1190	0.1191	0.1163	0.1127	0.0965	0.0965	0.0950	0.1027	0.1043	0.1062	0.1223	0.1147	0.1046	0.1046	0.1147	0.1303	0.1149	0.1566
NE0915	0.1270	0.1270	0.1052	0	0.0000	0.1168	0.1154	0.1155	0.1294	0.1275	0.1196	0.1213	0.1168	0.1227	0.1261	0.1263	0.1379	0.1292	0.1274	0.1274	0.1292	0.1563	0.1387	0.1797
NE0921	0.1270	0.1270	0.1052	0.0000	0	0.1168	0.1154	0.1155	0.1294	0.1275	0.1196	0.1213	0.1168	0.1227	0.1261	0.1263	0.1379	0.1292	0.1274	0.1274	0.1292	0.1563	0.1387	0.1797
NE1670	0.1145	0.1145	0.1136	0.1168	0.1168	0	0.1205	0.1190	0.1294	0.1275	0.1116	0.1133	0.1019	0.1214	0.1248	0.1211	0.1104	0.1264	0.1298	0.1298	0.1264	0.1375	0.1335	0.1852
NE0050	0.1279	0.1279	0.1190	0.1154	0.1154	0.1205	0	0.0028	0.1301	0.1265	0.1250	0.1267	0.1294	0.1232	0.1232	0.1234	0.1155	0.1233	0.1268	0.1268	0.1233	0.1594	0.1443	0.1660
NE0015	0.1281	0.1281	0.1191	0.1155	0.1155	0.1190	0.0028	0	0.1303	0.1266	0.1235	0.1252	0.1279	0.1213	0.1213	0.1218	0.1139	0.1215	0.1232	0.1232	0.1215	0.1574	0.1445	0.1622
NE1666	0.1369	0.1369	0.1163	0.1294	0.1294	0.1294	0.1301	0.1303	0	0.0028	0.1060	0.1077	0.1049	0.1171	0.1204	0.1295	0.1135	0.1092	0.1109	0.1109	0.1092	0.1515	0.1315	0.1858
NE0619	0.1333	0.1333	0.1127	0.1275	0.1275	0.1275	0.1265	0.1266	0.0028	0	0.1026	0.1042	0.1048	0.1135	0.1169	0.1259	0.1100	0.1057	0.1074	0.1074	0.1057	0.1478	0.1296	0.1841
NE0006	0.1244	0.1244	0.0965	0.1196	0.1196	0.1116	0.1250	0.1235	0.1060	0.1026	0	0.0014	0.0946	0.1175	0.1175	0.1076	0.1088	0.1040	0.1024	0.1024	0.1040	0.1228	0.1180	0.1634
NE0043	0.1261	0.1261	0.0965	0.1213	0.1213	0.1133	0.1267	0.1252	0.1077	0.1042	0.0014	0	0.0962	0.1192	0.1158	0.1093	0.1105	0.1057	0.1040	0.1040	0.1057	0.1211	0.1197	0.1652
NE1663	0.1181	0.1181	0.0950	0.1168	0.1168	0.1019	0.1294	0.1279	0.1049	0.1048	0.0946	0.0962	0	0.1084	0.1117	0.0973	0.1051	0.1075	0.1042	0.1042	0.1075	0.1319	0.1158	0.1714
NE0617	0.1303	0.1303	0.1027	0.1227	0.1227	0.1214	0.1232	0.1213	0.1171	0.1135	0.1175	0.1192	0.1084	0	0.0028	0.1111	0.1221	0.1054	0.1071	0.1071	0.1037	0.1300	0.1194	0.1595
NE0615	0.1303	0.1303	0.1043	0.1261	0.1261	0.1248	0.1232	0.1213	0.1204	0.1169	0.1175	0.1158	0.1117	0.0028	0	0.1111	0.1221	0.1087	0.1104	0.1104	0.1071	0.1266	0.1228	0.1631
NE1672	0.1366	0.1366	0.1062	0.1263	0.1263	0.1211	0.1234	0.1218	0.1295	0.1259	0.1076	0.1093	0.0973	0.1111	0.1111	0	0.0796	0.0949	0.0917	0.0917	0.0933	0.1071	0.1028	0.1661
NE1668	0.1330	0.1330	0.1223	0.1379	0.1379	0.1104	0.1155	0.1139	0.1135	0.1100	0.1088	0.1105	0.1051	0.1221	0.1221	0.0796	0	0.0858	0.0826	0.0826	0.0842	0.1116	0.0843	0.1673

NE0928	0.1340	0.1340	0.1147	0.1292	0.1292	0.1264	0.1233	0.1215	0.1092	0.1057	0.1040	0.1057	0.1075	0.1054	0.1087	0.0949	0.0858	0	0.0112	0.0112	0.0014	0.0896	0.0847	0.1585
NE0049	0.1323	0.1323	0.1046	0.1274	0.1274	0.1298	0.1268	0.1232	0.1109	0.1074	0.1024	0.1040	0.1042	0.1071	0.1104	0.0917	0.0826	0.0112	0	0.0000	0.0127	0.0944	0.0879	0.1621
NE0051	0.1323	0.1323	0.1046	0.1274	0.1274	0.1298	0.1268	0.1232	0.1109	0.1074	0.1024	0.1040	0.1042	0.1071	0.1104	0.0917	0.0826	0.0112	0.0000	0	0.0127	0.0944	0.0879	0.1621
NE0979	0.1340	0.1340	0.1147	0.1292	0.1292	0.1264	0.1233	0.1215	0.1092	0.1057	0.1040	0.1057	0.1075	0.1037	0.1071	0.0933	0.0842	0.0014	0.0127	0.0127	0	0.0879	0.0831	0.1567
NE1667	0.1409	0.1409	0.1303	0.1563	0.1563	0.1375	0.1594	0.1574	0.1515	0.1478	0.1228	0.1211	0.1319	0.1300	0.1266	0.1071	0.1116	0.0896	0.0944	0.0944	0.0879	0	0.1077	0.1728
NE0927	0.1477	0.1477	0.1149	0.1387	0.1387	0.1335	0.1443	0.1445	0.1315	0.1296	0.1180	0.1197	0.1158	0.1194	0.1228	0.1028	0.0843	0.0847	0.0879	0.0879	0.0831	0.1077	0	0.1561
NE1572	0.1966	0.1966	0.1566	0.1797	0.1797	0.1852	0.1660	0.1622	0.1858	0.1841	0.1634	0.1652	0.1714	0.1595	0.1631	0.1661	0.1673	0.1585	0.1621	0.1621	0.1567	0.1728	0.1561	0

S6-3: Wolbachia Tree:

```
(((ST_B10:0.213674,((ST_B8:0.047135,(ST_B3:0.034949,(ST_B9:0.030452,
((ST_B7:0.010367,ST_B6:0.010367):0.012933,
(ST_B2:0.006653,ST_B1:0.006653):0.016647):0.007152):0.008863):0.00661):0.014053,
(ST_B4:0.014958,ST_B5:0.014921):0.046257):0.175919):0.283651,(ST_A7:0.061632,
((ST_A5:0.022405,(ST_A3:0.011589,
(ST_A2:0.003006,ST_A4:0.003006):0.008584):0.010865):0.020511,
(ST_A1:0.011696,ST_A6:0.011696):0.031241):0.018695):0.567523):0.028739,
((ST_F8:0.014576,ST_F4:0.022147,(ST_F5:0.012127,ST_F7:0.013065):0.006443):0.145278,
(ST_F3:0.017176,ST_F1:0.017639):0.268154):0.327235);
```

S6-4: Wolbachia MLST genetic distance matrix:

	ST_B10	ST_B8	ST_B3	ST_B9	ST_B7	ST_B6	ST_B2	ST_B1	ST_B4	ST_B5	ST_A7	ST_A5	ST_A3	ST_A2	ST_A4	ST_A1	ST_A6	ST_F8	ST_F4	ST_F5	ST_F7	ST_F3	ST_F1
ST_B10	0	0.0426	0.0526	0.0630	0.1278	0.1229	0.0754	0.0851	0.0343	0.0406	0.0949	0.1285	0.1359	0.1367	0.1380	0.1276	0.1192	0.1342	0.1245	0.1112	0.1325	0.1354	0.1392
ST_B8	0.0426	0	0.0284	0.0412	0.1215	0.1167	0.0337	0.0508	0.0074	0.0222	0.0911	0.1215	0.1289	0.1350	0.1360	0.1163	0.1173	0.1447	0.1295	0.1239	0.1406	0.1436	0.1474
ST_B3	0.0526	0.0284	0	0.0305	0.0824	0.0869	0.0337	0.0274	0.0000	0.0196	0.0683	0.1226	0.1298	0.1359	0.1368	0.1171	0.1184	0.1021	0.0682	0.0827	0.1076	0.1384	0.1422
ST_B9	0.0630	0.0412	0.0305	0	0.0485	0.0442	0.0615	0.0530	0.0338	0.0460	0.0366	0.1216	0.1239	0.1315	0.1325	0.0860	0.1173	0.1032	0.0761	0.1037	0.1085	0.1348	0.1386
ST_B7	0.1278	0.1215	0.0824	0.0485	0	0.0039	0.0506	0.0349	0.1223	0.1194	0.0510	0.1460	0.1369	0.1532	0.1534	0.0696	0.1460	0.0504	0.0024	0.0506	0.0504	0.0909	0.0909
ST_B6	0.1229	0.1167	0.0869	0.0442	0.0039	0	0.0506	0.0391	0.1223	0.1146	0.0467	0.1460	0.1369	0.1532	0.1534	0.0696	0.1460	0.0547	0.0083	0.0549	0.0547	0.0900	0.0900
ST_B2	0.0754	0.0337	0.0337	0.0615	0.0506	0.0506	0	0.0006	0.0337	0.0337	0.0894	0.1264	0.1326	0.1387	0.1397	0.1199	0.1223	0.0805	0.0023	0.0534	0.0940	0.0894	0.0940
ST_B1	0.0851	0.0508	0.0274	0.0530	0.0349	0.0391	0.0006	0	0.0344	0.0481	0.0716	0.1275	0.1334	0.1395	0.1405	0.1207	0.1234	0.0529	0.0000	0.0350	0.0688	0.0974	0.1009
ST_B4	0.0343	0.0074	0.0000	0.0338	0.1223	0.1223	0.0337	0.0344	0	0.0000	0.0859	0.1226	0.1298	0.1359	0.1368	0.1171	0.1184	0.1565	0.1384	0.1258	0.1475	0.1425	0.1475
ST_B5	0.0406	0.0222	0.0196	0.0460	0.1194	0.1146	0.0337	0.0481	0.0000	0	0.0888	0.1226	0.1298	0.1359	0.1368	0.1171	0.1184	0.1404	0.1233	0.1198	0.1373	0.1371	0.1409
ST_A7	0.0949	0.0911	0.0683	0.0366	0.0510	0.0467	0.0894	0.0716	0.0859	0.0888	0	0.0530	0.0781	0.0875	0.0885	0.0298	0.0448	0.1065	0.0813	0.1071	0.1103	0.1366	0.1404
ST_A5	0.1285	0.1215	0.1226	0.1216	0.1460	0.1460	0.1264	0.1275	0.1226	0.1226	0.0530	0	0.0025	0.0034	0.0025	0.0350	0.0346	0.1460	0.1460	0.1460	0.0808	0.1364	0.0808
ST_A3	0.1359	0.1289	0.1298	0.1239	0.1369	0.1369	0.1326	0.1334	0.1298	0.1298	0.0781	0.0025	0	0.0074	0.0081	0.0585	0.0355	0.0840	0.1460	0.1531	0.0587	0.0967	0.0587
ST_A2	0.1367	0.1350	0.1359	0.1315	0.1532	0.1532	0.1387	0.1395	0.1359	0.1359	0.0875	0.0034	0.0074	0	0.0006	0.0670	0.0345	0.0696	0.1460	0.1444	0.0480	0.0852	0.0480
ST_A4	0.1380	0.1360	0.1368	0.1325	0.1534	0.1534	0.1397	0.1405	0.1368	0.1368	0.0885	0.0025	0.0081	0.0006	0	0.0677	0.0355	0.0696	0.1460	0.1444	0.0489	0.0862	0.0489
ST_A1	0.1276	0.1163	0.1171	0.0860	0.0696	0.0696	0.1199	0.1207	0.1171	0.1171	0.0298	0.0350	0.0585	0.0670	0.0677	0	0.0026	0.1532	0.1460	0.1544	0.1455	0.1069	0.1455
ST_A6	0.1192	0.1173	0.1184	0.1173	0.1460	0.1460	0.1223	0.1234	0.1184	0.1184	0.0448	0.0346	0.0355	0.0345	0.0355	0.0026	0	0.1460	0.1460	0.1460	0.1328	0.0792	0.1328

ST_F8	0.1342	0.1447	0.1021	0.1032	0.0504	0.0547	0.0805	0.0529	0.1565	0.1404	0.1065	0.1460	0.0840	0.0696	0.0696	0.1532	0.1460	0	0.0000	0.0429	0.0000	0.0366	0.0366
ST_F4	0.1245	0.1295	0.0682	0.0761	0.0024	0.0083	0.0023	0.0000	0.1384	0.1233	0.0813	0.1460	0.1460	0.1460	0.1460	0.1460	0.1460	0.0000	0	0.0000	0.0000	0.0562	0.0562
ST_F5	0.1112	0.1239	0.0827	0.1037	0.0506	0.0549	0.0534	0.0350	0.1258	0.1198	0.1071	0.1460	0.1531	0.1444	0.1444	0.1544	0.1460	0.0429	0.0000	0	0.0429	0.0832	0.0832
ST_F7	0.1325	0.1406	0.1076	0.1085	0.0504	0.0547	0.0940	0.0688	0.1475	0.1373	0.1103	0.0808	0.0587	0.0480	0.0489	0.1455	0.1328	0.0000	0.0000	0.0429	0	0.0551	0.0281
ST_F3	0.1354	0.1436	0.1384	0.1348	0.0909	0.0900	0.0894	0.0974	0.1425	0.1371	0.1366	0.1364	0.0967	0.0852	0.0862	0.1069	0.0792	0.0366	0.0562	0.0832	0.0551	0	0.0255
ST_F1	0.1392	0.1474	0.1422	0.1386	0.0909	0.0900	0.0940	0.1009	0.1475	0.1409	0.1404	0.0808	0.0587	0.0480	0.0489	0.1455	0.1328	0.0366	0.0562	0.0832	0.0281	0.0255	0

S6-5: Host-Wolbachia Matrix:

	ST_B10	ST_B8	ST_B3	ST_B9	ST_B7	ST_B6	ST_B2	ST_B1	ST_B4	ST_B5	ST_A7	ST_A5	ST_A3	ST_A2	ST_A4	ST_A1	ST_A6	ST_F8	ST_F4	ST_F5	ST_F7	ST_F3	ST_F1
NE0613	0	0	1	0	0	0	0	0	0	1	0	0	0	0	0	1	0	0	0	0	0	0	0
NE0638	0	0	1	0	0	0	0	0	0	1	0	1	0	0	0	1	0	0	0	0	0	0	0
NE0455	1	0	0	0	0	0	0	0	0	0	0	0	0	0	0	0	0	0	0	0	0	0	0
NE0915	0	0	0	0	0	0	0	0	0	0	0	0	0	0	1	0	0	0	0	1	0	0	0
NE0921	0	0	0	0	0	0	0	0	0	0	0	0	0	0	1	0	0	0	0	1	0	0	0
NE1670	0	0	0	0	0	0	0	0	0	0	0	0	0	0	0	0	0	0	0	0	1	0	0
NE0050	0	0	0	0	0	0	0	0	0	0	0	0	0	1	0	0	0	0	1	0	0	0	0
NE0015	0	0	0	0	0	0	0	0	0	0	0	0	1	0	0	1	0	0	1	0	0	0	0
NE1666	0	0	0	0	0	0	1	0	1	0	0	0	0	0	0	0	0	0	0	0	0	1	1
NE0619	0	0	0	0	0	0	0	1	0	0	0	0	1	0	0	1	0	0	0	0	0	0	0
NE0006	0	1	0	0	0	0	0	0	0	0	0	0	0	0	0	1	0	0	0	0	0	0	0
NE0043	0	1	0	0	0	0	0	0	0	0	0	0	0	0	0	1	0	0	0	0	0	0	0
NE1663	0	0	0	0	0	0	0	0	0	0	0	0	0	0	0	0	0	1	0	0	0	0	0
NE0617	0	0	0	0	0	0	0	0	0	1	0	0	0	0	0	0	0	0	0	0	0	0	0
NE0615	0	0	0	0	0	0	0	0	0	1	0	0	0	0	0	0	0	0	0	0	0	0	0
NE1672	0	0	0	0	0	0	0	0	0	0	0	0	0	0	0	0	0	1	0	0	0	0	0
NE1668	0	0	0	0	0	0	0	0	0	0	0	0	0	1	0	0	1	0	1	0	0	0	0

NE0928	0	0	0	0	0	1	0	0	0	0	0	0	0	0	0	1	0	0	0	0	0	0	0
NE0049	0	0	0	0	1	1	0	0	0	0	0	0	0	0	0	1	0	0	0	0	0	0	0
NE0051	0	0	0	0	0	0	0	0	0	0	0	0	0	0	0	1	0	0	0	0	0	0	0
NE0979	0	0	0	0	0	0	0	0	0	0	0	0	0	0	0	1	0	0	0	0	0	0	0
NE1667	0	0	0	0	0	0	0	0	0	0	1	0	0	0	0	0	0	0	0	0	0	0	0
NE0927	0	0	0	1	0	0	0	0	0	0	0	0	0	0	0	1	0	0	0	0	0	0	0
NE1572	0	0	0	0	0	0	0	0	0	0	0	0	0	0	0	1	0	0	0	0	0	0	0

S6-6: Input file for Jane:

```
HOSTTREE
1     null
2     null
3     1     2
4     null
5     3     4
6     null
7     null
8     6     7
9     null
10    null
11    null
12    10    11
13    5     8     9     12
14    null
15    null
16    14    15
17    null
18    null
19    17    18
20    null
21    19    20
22    16    21
23    null
24    null
25    23    24
26    13    22    25
27    null
28    26    27
29    null
30    28    29
31    null
32    null
33    null
34    32    33
```

35	null		
36	31	34	35
37	null		
38	36	37	
39	30	38	
40	null		
41	39	40	
42	null		
43	41	42	

HOSTNAMES

1	NE0613
2	NE0638
3	3
4	NE0455
5	5
6	NE0915
7	NE0921
8	8
9	NE1670
10	NE0050
11	NE0015
12	12
13	13
14	NE1666
15	NE0619
16	16
17	NE0006
18	NE0043
19	19
20	NE1663
21	21
22	22
23	NE0617
24	NE0615

25	25
26	26
27	NE1672
28	28
29	NE1668
30	30
31	NE0928
32	NE0049
33	NE0051
34	34
35	NE0979
36	36
37	NE1667
38	38
39	39
40	NE0927
41	41
42	NE1572
43	43

PARASITETREE

44	null	
45	null	
46	null	
47	null	
48	null	
49	null	
50	48	49
51	null	
52	null	
53	51	52
54	50	53
55	47	54
56	46	55
57	45	56

58	null		
59	null		
60	58	59	
61	57	60	
62	44	61	
63	null		
64	null		
65	null		
66	null		
67	null		
68	66	67	
69	65	68	
70	64	69	
71	null		
72	null		
73	71	72	
74	70	73	
75	63	74	
76	62	75	
77	null		
78	null		
79	null		
80	null		
81	79	80	
82	77	78	81
83	null		
84	null		
85	83	84	
86	82	85	
87	76	86	

PARASITENAMES

44	ST_B10
45	ST_B8
46	ST_B3

47	ST_B9
48	ST_B7
49	ST_B6
50	50
51	ST_B2
52	ST_B1
53	53
54	54
55	55
56	56
57	57
58	ST_B4
59	ST_B5
60	60
61	61
62	62
63	ST_A7
64	ST_A5
65	ST_A3
66	ST_A2
67	ST_A4
68	68
69	69
70	70
71	ST_A1
72	ST_A6
73	73
74	74
75	75
76	76
77	ST_F8
78	ST_F4
79	ST_F5
80	ST_F7
81	81
82	82

83	ST_F3
84	ST_F1
85	85
86	86
87	87

PHI

4	44			
17	45	71		
1	46	59	71	
40	47	71		
32	48	71	49	
31	49	71		
14	51	58	83	84
15	52	71	65	
14	51	58	83	84
1	46	59	71	
37	63			
2	64	71	59	46
11	65	78	71	
10	66	78		
6	67	79		
1	46	59	71	
29	72	78	66	
20	77			
10	66	78		
6	67	79		
9	80			
14	51	58	83	84
14	51	58	83	84
7	79	67		
27	77			
18	71	45		
33	71			
35	71			

42	71
24	59
23	59

HOSTRANKS

1	1
2	1
3	1
4	1
5	1
6	1
7	1
8	1
9	1
10	1
11	1
12	1
13	1
14	1
15	1
16	1
17	1
18	1
19	1
20	1
21	1
22	1
23	1
24	1
25	1
26	1
27	1
28	1
29	1
30	1

31	1
32	1
33	1
34	1
35	1
36	1
37	1
38	1
39	1
40	1
41	1
42	1
43	1

PARASITERANKS

44	1
45	1
46	1
47	1
48	1
49	1
50	1
51	1
52	1
53	1
54	1
55	1
56	1
57	1
58	1
59	1
60	1
61	1
62	1
63	1

64	1
65	1
66	1
67	1
68	1
69	1
70	1
71	1
72	1
73	1
74	1
75	1
76	1
77	1
78	1
79	1
80	1
81	1
82	1
83	1
84	1
85	1
86	1
87	1

S6-7: R scripts used for PACo and ParaFit analyses:

```
#This script was adapted in part from Hutchinson et al. (2017).

#paco: implementing Procrustean Approach to Cophylogeny in R. Meth. Ecol. Evol.8(8): 932-940.


#Load the required packages


library(paco)

library(ape)

library(ggplot2)
```

```
##### Procrustean Approach to Cophylogeny (PACo)

#Read genetic distance matrixes of hosts (damselflies)
#and Wolbachia strands and a matrix that represent the interactions
#between hosts and Wolbachia

h<-read.table("Host-matrix.txt", header = T, dec = ".")
p<-read.table("Parasite-matrix.txt", header = T, dec = ".")
hp<-read.table("HP-matrix.txt", header = T, dec = ".")

##############################################################################
#Test the hypothesis that Wolbachia phylogeny and hosts phylogeny depend on
each other#
##############################################################################

D1 <- prepare_paco_data(h, p, hp) # Bundle distance matrixes and interaction
matrix
D1 <- add_pcoord(D1, correction = "cailliez") # perform Principal Coordinate
analysis

# Perform cophylogenetic analysis and show the results
# of a goodness-of-fit test.

D1<- PACo(D1, nperm=1000, seed=1, method='quasiswap', symmetric = T,
proc.warnings = F)
residual_sum_of_squares1<-(D1$gof$ss)
p_value1<-(D1$gof$p)
print(goftest1<-cbind(residual_sum_of_squares1, p_value1))

#Plot a tangelgram depicting the associations between host species and
parasites
# to weight the interactions we use the cophylogenetic contribution
(Procrustes squared residuals) transformed to best show
# the differences graphically
```

```
hosttree<-read.tree("Host-COI-prunned.tre")

parasttree<-read.tree("Wolbachia-prunned.tre")


res1 <- residuals_paco(D1$proc)

weight1 <- (res1^-2)/50

assoc <- data.frame(host=rownames(hp)[which(hp==1, arr.ind=TRUE)[,'row']],
par=colnames(hp)[which(hp==1, arr.ind=TRUE)[,'col']])

cophyloplot( hosttree,parasttree,assoc, type= "cladogram",lwd=weight1,
use.edge.length=FALSE, col= "steelblue", length.line= 0, gap=1, space=50)


# return interaction-specific cophylogenetic contributions

# based on a jackknife procedure and plot the results

Djck1 <- paco_links(D1)

upperjack1<-as.vector(Djck1$jackknife$upper)

Squared_residuals<-as.vector(Djck1$jackknife$mean)

median<-median(Squared_residuals)

Host_Wolbachia_Links<-c("NE0455-ST_B10", "NE0006-ST_B8" ,"NE0043-ST_B8",
"NE0613-ST_B3",

                        "NE0638-ST_B3",  "NE0927-ST_B9", "NE0049-ST_B7",
"NE0928-ST_B6",  "NE0049-ST_B6",

                        "NE1666-ST_B2",  "NE0619-ST_B1",  "NE1666-
ST_B4" ,"NE0613-ST_B5", "NE0638-ST_B5",

                        "NE0617-ST_B5",  "NE0615-ST_B5",  "NE1667-
ST_A7"  ,"NE0638-ST_A5",  "NE0015-ST_A3",

                        "NE0619-ST_A3",  "NE0050-ST_A2",  "NE1668-
ST_A2"  ,"NE0915-ST_A4",  "NE0921-ST_A4",

                        "NE0613-ST_A1",  "NE0638-ST_A1",  "NE0015-
ST_A1"  ,"NE0619-ST_A1",  "NE0006-ST_A1",

                        "NE0043-ST_A1",  "NE0928-ST_A1",  "NE0049-
ST_A1" ,"NE0051-ST_A1",  "NE0979-ST_A1",

                        "NE0927-ST_A1",  "NE1572-ST_A1",  "NE1668-
ST_A6"  ,"NE1663-ST_F8", "NE1672-ST_F8",

                        "NE0050-ST_F4",  "NE0015-ST_F4",  "NE1668-
ST_F4" ,"NE0915-ST_F5",  "NE0921-ST_F5",

                        "NE1670-ST_F7",  "NE1666-ST_F3",  "NE1666-ST_F1")

data <- data.frame(Host_Wolbachia_Links,Squared_residuals,upperjack1)

df <- data.frame(x1 = 0, x2 = 48, y1 = median, y2 = median)

ggplot(data) +

  geom_bar( aes(x=Host_Wolbachia_Links, y=Squared_residuals), stat="identity",
fill="gray") +
```

```
  geom_errorbar( aes(x=Host_Wolbachia_Links, ymin=Squared_residuals,
ymax=Squared_residuals+upperjack1), width=0.5, colour="black", alpha=0.9,
size=1) +

  geom_segment(aes(x = x1, y = y1, xend = x2, yend = y2), data = df)+

  coord_flip() + theme_bw() + theme(panel.grid = element_blank(),
panel.border=element_blank(),axis.ticks.y = element_blank(), axis.line.x=
element_line(colour = "black")) +

  ylab("Squared residuals") + xlab("Host-Wolbachia Links")



##############################################################################
################################

#ParaFit analysis. Test the null hypothesis that the host taxa and Wolbachia
strands are randomly associated.#

#Additionally, tests the relative contribution of individual associations to
the overall congruence.         #

##############################################################################
################################



res_parafit <- parafit(h, p, hp, nperm=1000, correction = "cailliez",
test.links=TRUE)

ParaFitGlobal<-(res_parafit$ParaFitGlobal)

p.global<-(res_parafit$p.global)

print(Globaltest<-cbind (ParaFitGlobal, p.global))

print(res_parafit$link.table)
```
